# Gangliosides as Biomarkers of Human Brain Diseases: Trends in Discovery and Characterization by High-Performance Mass Spectrometry

**DOI:** 10.3390/ijms23020693

**Published:** 2022-01-08

**Authors:** Mirela Sarbu, Raluca Ica, Alina D. Zamfir

**Affiliations:** 1 Department of Condensed Matter, National Institute for Research and Development in Electrochemistry and Condensed Matter, Plautius Andronescu Str. 1, 300224 Timisoara, Romania; mirela.sarbu86@yahoo.co.uk (M.S.); raluca.ica@gmail.com (R.I.); 2 Department of Physics, West University of Timisoara, Vasile Parvan Blvd. 4, 300223 Timisoara, Romania; 3 Department of Technical and Natural Sciences, “Aurel Vlaicu” University of Arad, Revolutie Blvd. 77, 310130 Arad, Romania

**Keywords:** gangliosides, mass spectrometry, brain diseases, biomarkers, structural analysis

## Abstract

Gangliosides are effective biochemical markers of brain pathologies, being also in the focus of research as potential therapeutic targets. Accurate brain ganglioside mapping is an essential requirement for correlating the specificity of their composition with a certain pathological state and establishing a well-defined set of biomarkers. Among all bioanalytical methods conceived for this purpose, mass spectrometry (MS) has developed into one of the most valuable, due to the wealth and consistency of structural information provided. In this context, the present article reviews the achievements of MS in discovery and structural analysis of gangliosides associated with severe brain pathologies. The first part is dedicated to the contributions of MS in the assessment of ganglioside composition and role in the specific neurodegenerative disorders: Alzheimer’s and Parkinson’s diseases. A large subsequent section is devoted to cephalic disorders (CD), with an emphasis on the MS of gangliosides in anencephaly, the most common and severe disease in the CD spectrum. The last part is focused on the major accomplishments of MS-based methods in the discovery of ganglioside species, which are associated with primary and secondary brain tumors and may either facilitate an early diagnosis or represent target molecules for immunotherapy oriented against brain cancers.

## 1. Introduction

Gangliosides (GGs) represent a particular class of glycosphingolipids with a complex structure, ubiquitously identified in tissues and body fluids, and highly expressed in the central nervous system (CNS), in particular in various brain regions [1,2,3]. Embedded in the outer leaflet of the plasma membrane, the ceramide lipid tail consisting of long-chain bases (sphingosine) and fatty acyl chains of different lengths and degrees of saturation [4,5] is glycosidically linked to a glycan headgroup containing one or more sialic acid residues. Extended into the extracellular environment, the glycan core, especially through the terminal sialic acid residues [6], participates in specific and essential biological functions of the brain [3], such as cell-to-cell recognition/communication and signaling, modulating, or triggering a variety of biological events, including those related to brain development, maturation, and aging [7,8,9,10]. GGs are also strongly correlated with brain disorders through aberrant glycosylation pathways. Remarkably, GGs account for about 80% of all glycan mass and over 75% of the sialic acid present in the brain [11].

**Scheme 1:**
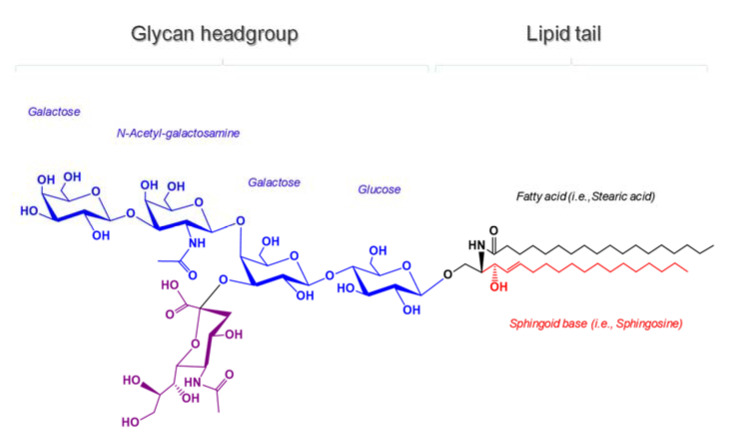
Structure of GM1, one of the major ganglioside components of human brain.

At the nervous system level, the expression, distribution, and structure of GGs are cell and tissue type-specific and vary during tissue development, maturation, aging, and most of all, in pathological states [12,13]. An increase in GG content and degree of sialylation caused by changes in the expression level and activity of the enzymes implicated both in the synthesis and degradation of GGs was observed. Thus, the level of GM3 and GD3, the major GGs in the human embryonic brain, is decreased with the concomitant increase in more complex GGs, such as GM1 (Scheme 1), GD1a, GD1b, and GT1b [5,14], which represent over 90% of the GG mass in the adult brain.

The 10 to 30 times higher GG content in the brain than in any other tissue or organ in the body, as well as making the brain the most vulnerable organ if any changes in GG composition and quantity occur, highlights the particular role of GGs in the CNS. Hence, GGs represent important diagnostic biomarkers of neurodegenerative disorders and malignant transformations [15], for which these molecules were demonstrated to act as both inhibitors and stimulators. More importantly, GGs are also considered therapeutic targets to be further studied for vaccine and/or drug development [16].

In this context, a comprehensive and clear mapping and structural elucidation of individual GG components are therefore of critical necessity not only for a proficient characterization of the nervous system-specific GG components in health and disease but also for correlating the composition and structure specificity with the pathologies affecting the CNS.

In view of the overwhelming effects and incidence of neurodegenerative disorders, as well as of benign and malignant primary and secondary nervous system tumors, in this review we assess the state-of-the-art in the implementation of the most modern mass spectrometry techniques and related protocols in the research focused on GG biomarker discovery in brain diseases. As highlighted here, in recent years, MS not only became a high sensitivity analytical tool indispensable for clinical assays targeting brain GGs and the discovery of specific biomarkers but also has developed into a method that can be used to follow the evolution of the disease and the response to the treatment.

## 2. Neurodegenerative Diseases

Neurodegenerative diseases represent a group of incurable ailments characterized by the degeneration of the structure and function of the CNS or peripheral nervous system. The essential role of GGs in CNS homeostasis is highlighted by the fact that both a lack and an excess of GGs cause severe neurodegenerative disorders. While accumulation of GGs caused by mutations in lysosomal degradative enzymes determines deadly lysosomal storage diseases, among which are GM1-gangliosidosis and Tay–Sachs and Sandhoff diseases, the lack of GG synthesis results in severe early onset epileptic syndromes and neurodegeneration [11,17]. Changes in the GG profile were also reported in some common neurological conditions, such as Huntington’s disease (HD) [18,19], Alzheimer’s disease (AD) [20,21,22], Parkinson’s disease (PD) [23,24,25,26], amyotrophic lateral sclerosis [27], stroke [28], multiple sclerosis [29,30], and epilepsy [31]. 

While for many conditions, including cancer [32], diabetes [33], and infection [34], the key role of GGs is recognized and effective treatments have been identified, unfortunately, for many degenerative neurological diseases no reported progress has so far been reported in this direction [35].

### 2.1. Alzheimer’s Disease

AD, an irreversible, gradually progressive neurodegenerative disease is the most prevalent and devastating form of dementia worldwide among people over age 65, which ultimately causes death. Conversion of the soluble, nontoxic amyloid β-protein (Aβ) into an aggregated, toxic form rich in β-sheets, as well as aggregation of hyperphosphorylated microtubule protein tau in neurofibrillary tangles, are the pathological hallmarks of AD [11]. Aβs are able to bind to a variety of membrane components, such as cholesterol [36,37,38], phospholipids [39], and gangliosides [40,41,42].

Given the vital neuroprotective roles of brain GGs, as well as the neuritogenic and neuronotrophic activity and the ability of GM1 to repair the neuronal tissue after mechanical, biochemical, or toxic injuries, GM1 was proposed almost three decades ago as a therapeutic agent in AD [43]. However, with all the research in the field, abnormal GG expression was directly connected to AD, but the exact GG involvement in AD remained controversial. While the complex of GM1 and Aβ, termed GAβ, identified in AD brains seems to act as an endogenous seed for the Aβ fibrillogenesis in the AD brain [44,45,46,47,48], administration of intraventricular GM1 resulted in a reduction in Aβ level in the brain and marked improvements in several clinical outcomes, which suggests that GM1 might serve as a therapeutic agent [49]. On the other hand, recent studies [50,51] substantiated that, actually, GAβ represents the starting point for the formation of Aβ aggregates and discovered that not only the association with the membrane but also the subsequent membrane perforation induced by Aβ is determined by certain levels of GM1. Consequently, altogether, the findings suggest so far that depending on the level of expression, GM1 might play a role in either the association of Aβ with the membrane and the formation of pores leading to membrane breakage or, oppositely, in the reduction in Aβ in the brain.

Over the years, several papers reported not only that the degree of GG alteration is directly influenced by the age of AD onset [52] but also that it is topographically dependent [53,54]. A decreased concentration of GM1, GD1a, GD1b, and GT1b in a region-specific manner, [52,55,56] and an increase in simple GG glycoforms such as GM2, GM3, and GM4 in the frontal cortex of patients was documented [55,56]. Contrastingly, Brooksbank and McGovern [57] as well as Crino et al. [58] revealed a slight increase in GM1, GT1a, GD3, and GM2, therefore suggesting the direct correlation within an abnormal GG metabolism and the affected cortical areas of neurodegeneration that afflicts AD.

With a pivotal role in cholinergic synaptic transmission and cognitive function [59], cholinergic-specific antigen-1α (Chol-1α) antigens were reported in sera of AD [60,61]. Ariga et al. showed an increase inthe Chol-1α antigens GQ1bα and GT1aα in the brain of a transgenic mouse AD model [62] and a predilection of Aβ1–40 for binding to a number of GGs with the following order of binding strength: GQ1bα > GT1aα > GQ1b > GT1b > GD3 > GD1a = GD1b > LM1 > GM1 > GM2 = GM3 > GM4, based on surface plasmon resonance studies [63]. On the other hand, specific monoclonal antibodies approaches highlighted the incidence of GM1 [64], c-series GGs [65], and GD1a [66] in senile plaques and the possibility of Aβs interaction with them.

If the neuronal development and regeneration are GG-dependent processes, the anti-GG antibodies display a reverse activity. Hence, their presence, especially of anti-GM1 antibody, in different biological matrices was used by some researchers as brain biomarkers and was associated with neuronal degeneration in AD and PD with dementia [67,68]. Measuring by enzyme-linked immunosorbent assay (ELISA), the serum levels of anti-GG antibodies in patients with diagnosed AD and vascular dementia and comparing them to sera of normal controls, Miura et al. [69] demonstrated that titers of IgG and IgM anti-GM1, anti-GQ1bα, and anti-GT1aα antibodies did not differ among the three investigated matrices. Consequently, according to Miura et al., the anti-GG antibodies are improbable to be pathogenic in AD or to be useful as biomarkers [69].

More recent findings disclose the increase in GGs with long chain bases (d20:1) in the hippocampus and other affected brain regions of AD mice, which may document their implication in AD dysfunctions [70,71,72]. In this context, several MS-based approaches contributed to significant progress in the field. By developing a new method based on a matrix-assisted laser desorption/ionization—time of flight (MALDI-TOF) MS system in combination with a method for transferring lipids separated on a TLC-plate to a poly-vinylidene difluoride membrane and direct mass spectrometric analysis of the individual lipids on the membrane, Taki [73] demonstrateda lower expression of the b-series gangliosides GD1b and GT1b in the hippocampal gray matter of patients with AD than those in patients with Parkinson’s disease or the control. The molecular scanning of a single GG molecular species demonstrated a considerable reduction in species exhibiting (d20:1/18:0) ceramide composition in patients with AD [73]. Another remarkable example illustrating the efficiency of the MS method, in particular when combined with liquid chromatography (LC), belongs to Oikawa et al. [20]. In 2015, the authors observed an increase in the ratio of the level of GD1b-ganglioside containing C20:0 fatty acid to that containing C18:0 in specimens of precuneus and calcarine cortex from human brains neuropathologically classified and diagnosed with AD. 

Additionally, MALDI imaging MS was demonstrated as an efficient analytical platform for evaluating the expression and levels of a-series GG species GD1a, GM1, GM2, and GM3 in a combined rat model of Aβ toxicity and stroke. In the study of Caughlin et al. [74], MALDI imaging MS revealed for the first time an alteration in GG composition in stroke and a different variation of their expression when Aβ toxicity was present as a comorbidity. From the clinical viewpoint, these results suggest for the first time that a synergic molecular mechanism might be involved in brains affected by AD and stroke comorbidities. Another remarkable finding is related to the expression of d18:1 and d20:1 species. Hence, the significant changes in expression observed in the d20:1 species that did not reach statistical significance in the d18:1 species suggest future therapeutics targeting GGs, as the d20:1 form may prove to be a more effective therapeutic target than the d18:1 species [74].

Considering the spatial arrangement of GM1 expressed in the hippocampus, the region strongly affected by AD, Hirano-Sakamaki et al. [71] investigated by imaging MS the mechanism of dysfunction that occurs in AD. A remarkable decrease in the ratio of GM1(d20:1/18:0) to GM1(d18:1/18:0) in the outer molecular layer of the dentate gyrus, which is to be correlated with the neurodegeneration that characterizes this particular form of dementia, was discovered. Similarly, in a previous study based on thin layer chromatography (TLC) blot/MALDI MS, the authors also found AD-related decreases in the ratio of d20:1-ganglioside to d18:1-ganglioside on GM1, GD1a, GD1b, and GT1b in the hippocampal gray matter of AD patients [75].

The detailed investigation by MALDI imaging MS of GM1 involvement in AD [76] additionally revealed an altered GM1 to GM2/GM3 ganglioside metabolism in the diffuse periphery of amyloid plaques but not in the core region and an enrichment of Aβ1-40arc peptide at the core of these deposits. The results highlight that GM1 is rather localized in the mature amyloid structure, interacts with the proteins at this level, and is greatly involved in AD pathology via such interaction mechanisms.

In addition to these alterations in GG profile, other important changes in GG levels that occur in AD, PD, and HD, as well as their contribution to cellular dysfunctions and disease pathogenesis, were recently comprehensively reviewed by Touboul and Gaudin [77] and by Sippne at al. [11].

### 2.2. Parkinson’s Disease

PD is the second most common, multifactorial, chronic, and progressive neurodegenerative disorder after AD, in which patients experience motor and non-motor deficits due to degeneration of dopaminergic neurons mainly in the substantia nigra and fibrillar cytoplasmic aggregation of α-synuclein [11]. Currently, the exact pathogenesis of PD is uncertain; while mutations in genes account for only 10% of PD occurrences, aging, environmental factors (i.e., exposure to pesticides, metals, and manganese), oxidative stress, mitochondrial dysfunction, cellular senescence, and epigenetic alterations contribute as well to the etiology of PD [78,79,80,81]. Clinically, bradykinesia, rigidity, resting tremor, gait disturbance, and postural instability as well as cognitive, affective, and autonomic components were found to characterize PD [35]. Nowadays, there is no cure for PD or drug to stop the progression of the disease, only treatments to manage symptoms.

Currently, the small oligomers of α-synuclein are considered the primary neurotoxic species in PD. Among such oligomers, those forming pores in the plasma membrane of brain cells exhibit the highest toxicity since such species favor the penetration of Ca^2+^ ions in massive amounts. The investigation of GG involvement in the toxicity of these oligomers has recently confirmed that gangliosides are relevant therapeutic targets for both AD and PD [82]. On the other hand, the ability of GM1 to protect against α-synuclein toxicity in vivo has also been postulated. A recent report has suggested that GM1 administration protects against the loss of dopamine neurons from the substantia nigra and from reduction in striatal dopamine levels. At the same time, GM1 was shown to reduce α-synuclein aggregation [83]. Hence, the neurodegeneration associated with PD is clearly dependent on the GG content; α-synuclein is a GM1-binding protein [25,84], whereas GM2, GM3, and asialo-GM1 exhibit a weaker bound on α-synuclein [85].

In the context in which the GGs concentration plays a critical role in PD development, since through this bindingof the fibrillation of α-synuclein and its pathological accumulation can be avoided, important efforts were invested into the development of high-performance biophysical methods able to provide better insight into the modifications that occur in the glycolipid pattern with PD progression. Various studies have related altered GG levels and parkinsonism both in mouse models and patients. While a partial decrease in the GG levels in mice determined short-term memory loss and heart and colon lesions, as well as signs of constipation, all being specific for PD [86,87,88], the administration of various drugs, such as L-DOPA, GM1, or the semisynthetic lysoderivative of GM1, Liga20, diminished symptoms and neuropathology and increased α-synuclein clearance either by stimulating autophagy or preventing its aggregation [83,87,88,89,90]. Compared with healthy subjects, reduced levels of GD1a and GT1b [91], as well as decreased levels of GM1 and GD1b, and a concomitant increase in the GG precursor glucosylceramide [92] were reported in the substantia nigra of PD patients. Similar results were also observed in PD cerebrospinal fluid (CSF) samples, where the total GG levels, particularly of GM2, GD3, GD1a, GD1b, and GT1b, were considerably decreased, and in PD serum, where just GM1 and GD1a were reduced [92]. Moreover, elevated levels of IgM antibodies against GM1, GD1b, and GQ1b gangliosides in the sera of patients who suffered from idiopathic PD vs. healthy age-matched individuals were also described [85]. 

High-performance mass spectrometry effectively contributed to the investigation of the plasma lipidome of PD patients. Hence, in 2017, Zhang et al. [93] substantiated the biomarker role of GM3 and the significantly higher GM3 plasma concentration in PD patients. In high-performance liquid chromatography (HPLC) MS-derived quantitative lipidomics studies on the plasma profile obtained from 170 PD patients vs. 120 controls, major differences between PD patients and controls were observed in the case of GM3 (ganglioside-NANA-3) concentration (1.293 ± 0.029 pmol/μL vs. 1.488 ± 0.041 pmol/μL, respectively) after normalization with respect to the total lipid content. Testing 39 subclasses of plasma lipids, including 520 specific lipid species in 150 PD cases and 100 controls by using the same methodology based on HPLC MS, Chan et al. [94] obtained similar results on GM3 concentration in PD vs. controls (1.531 ± 0.037 pmol/μL vs. 1.337 ± 0.040 pmol/μL, respectively) as Zhang et al. [93]. Moreover, this discrepancy in GM3 levels was stronger in males than in females. Therefore, these two analyses not only documented on one side the clear association of PD with higher plasma GM3 levels and on the other side the potential role of GM3 as a biomarker for idiopathic PD but also opened further directions for investigating the interactions between GM3 and α-synuclein. 

PD was also approached using ultra performance liquid chromatography (UPLC) MS and the tandem MS (MS/MS) method, which was developed, optimized, and validated for multiplex analysis of GlcCer isoforms (C18:0, C20:0, C22:0, C24:1, and C24:0) in brain tissue samples in order to determine whether GCase deficiency was associated with the accumulation of its glucosylceramide (GluCer) substrate in PD brain tissues [95]. The temporal cortex brain samples were collected postmortem from 26 PD patients in different (IIa, III, and IV) stages of the disorder and compared with brain tissue samples from 12 controls and 6 patients with incidental Lewy body disease. The normal phase UPLC separation of the total lipid extract allowed the discrimination of GlcCer and GalCer isobaric structures, a necessary step given that GalCer isoform concentration is about 300 times higher than that of GluCer. However, the further online detection by multiple reaction monitoring (MRM) MS and structural analyses by tandem MS of GluCer isoform revealed no significant GluCer concentration differences between the Parkinson disease sample groups and the control group tissues [95].

Starting from the discovery that the free soluble oligosaccharide of GM1 is the bioactive portion of GM1 for neurotrophic functions, an interesting approach for a new human therapy of PD was recently proposed by Chiricozzi et al. [96]. Following 4 weeks of administrating the soluble oligosaccharide of ganglioside GM1 (20 mg/kg) to heterozygous B4galnt1^+^/^−^ mice, a model of sporadic PD, the GG pattern and content in the cortex and cerebellum was determined by HPLC and ion trap MS. The results have shown that GM1 oligosaccharide reaches the brain and completely rescues the physical symptoms, reduces the aberrant levels of α-synuclein in the substantia nigra pars compacta (SNpc), and restores nigral tyrosine hydroxylase expression and striatal neurotransmitter levels, overlapping the wild-type condition. Though their study does not explain the exact mechanism by which the oligosaccharide moiety of GM1 rescues the PD phenotype, a hypothesis is that the reduced level of GM1, and hence of its oligosaccharide, triggers the neurodegenerative process by a failure in trophic signals, together with a reduction in α- synuclein clearance [96]. 

Altogether, the results presented above propose GM1 as a disease-modifying therapy for PD, being able to avert aggregation of endogenous α-synuclein and endorse its cellular clearance.

## 3. Cephalic Disorders

Cephalic disorders (CD), also known as neurodevelopmental disorders, represent a series of congenital conditions that affect the structure and function of the human brain and CNS, including the skull growth. The incidence of the CDs that affect infants and children worldwide is either due to abnormalities occurring in the very early stages of a child’s CNS and brain development in uterus, or, to a lesser extent, due to trauma to the fetus.

The exact cause of CDs is still unknown. Experts consider that hereditary or genetic conditions are the main factor; however, they do not exclude that maternal nutritional deficiency and exposure of the mother and developing fetus to infections, alcohol, cigarettes, illegal drugs, toxic substances, medications or radiation, as well as a reduced intake of folic acid during pregnancy, may also play a role in developing CDs [97,98].

Since in some CDs, the cranial sutures join prematurely, the abnormal shape or size of a baby’s head is one of the most noticeable signs of a CD. Depending on the affected brain areas and degree, the symptoms may range from mild to severe, as systematized in Table 1. While many people with CD have a relatively normal life, others may present developmental delays, physical disabilities, or even premature death in the form of miscarriage or stillbirth. While some CDs are present and unmistakably detected at birth, others are not. The same range is present for their treatment: some may be treated with medications, physical/speech therapy, or surgery to help correct a deformed skull or face, while some CDs cannot be treated, with such patients having a usually poor prognosis. 

In order to understand what triggers the CDs, it is extremely important first to decipher the chemical composition of the affected CNSs and then to identify the cause of the changes by comparing the data with the research collected on the normal development of the human nervous system. It is generally accepted that: (i) GGs, as vital components of neuronal cell membranes, play a critical role in neuronal function and brain development [13,127,128] and (ii) dramatic consistent changes in brain GG expression not only occur but also are region-specific [11,129,130], being closely regulated with neurodevelopmental stages, including neural tube formation [127,131,132]. Hence, any loss-of-function mutations in genes that encode GG biosynthetic enzymes lead to serious neurodevelopmental and neurodegenerative diseases [31,133,134,135]. A recent review [11] not only summarizes the crucial functions of GGs to maintain brain health, as well as the changes in GGs levels that occur in major neurological conditions, but also tries to answer the question of whether the interventions that restore normal GG levels could be neuroprotective. 

Regarding the composition and role of GGs in the onset and/or diagnosis of CDs, there are few studies. By investigating the amniotic fluid of pregnant women infected with Zika virus (ZIKV), the group of Morrot [136] demonstrated recently that the infection is associated with severe neurological manifestations of microcephaly on the fetus, as the ZIKV can cross the placental barrier. Although the microcephaly is the main and widespread reported clinical manifestation in fetuses delivered by mothers infected with ZIKV during pregnancy, anencephaly cases are also allied with ZIKV infection at incipient stages of fetal development [137]. By using ELISA, they tested the serum of patients with Zika infection for anti-GG antibodies and demonstrated a significant increase in the levels of total IgG autoantibodies, recognizing brain GGs in the sera of Zika-infected patients. Moreover, the screening of all patients and healthy controls sera for IgG subclasses against GD1a, GD3, GM2, GD1b, and GT1b human brain GGs revealed in Zika-infected patients elevated levels of IgG autoantibodies against GD3, a class of glycolipid highly expressed in neural stem cell. Since GD3 and GM3 are predominant in the embryonic brain and represent the building blocks for more complex and closely related forms, such as GM1a, GD1a, GD1b, and GT1b, the binding of autoantibodies to GD3 causes a homeostatic imbalance of neural cells, affecting neurogenesis during embryonic/fetal development. In view of the current results, the authors recommend GD3 as a biomarker to identify patients at risk of developing autoimmune complications in the context of ZIKV infections [136].

Anencephaly, the most common and severe disease in the CD spectrum and determined by a neural tube closure defect, is characterized by the absence of the cerebral hemispheres and the cranial vault, incomplete brain development, and missing cranial bones above the orbits [138]. The anencephalic cerebral remnant represents for more than two decades a model to explore GG involvement in triggering abnormal brain development. Since 2001, several approaches based on advanced MS were conducted for identification and fragmentation analysis of anencephaly-associated GG species.

In 2001, Vukelić et al. [1] employed TLC, fast atom bombardment (FAB) MS, nanoelectrospray ionization (nanoESI) quadrupole time-of-flight (QTOF) MS and tandem MS for compositional and structural determination of native GGs from two anencephalic cerebral residues from fetuses in the 30 and 37 gestational weeks. Their approach revealed a total GG concentration that was 34% and 13% lower in the anencephalic cerebral remnant and in cerebellum, respectively, in relation to the age-matched controls. Moreover, in the cerebral remnant, GD3, GM2, GT1b, GM1b nLM1, and nLD1 were highly expressed, and GD1a was decreased in the anencephalic cerebral remnant but enriched in anencephalic cerebellum, while GQ1b was reduced in both anencephalic regions [1]. If this study provided mainly quantitative data, the extended MS investigation carried out in 2008 by employing fully automated chip nanoESI high capacity ion trap (HCT) MS and multistage MS (MS^n^) by collision-induced dissociation (CID) provided important information related to the GG expression in anencephalic vs. age-matched normal brain tissue [139,140]. Here, the comparison of the GG mixture extracted from glial islands of anencephalic residual brain tissue in the 28th gestational week (An28) with the GGs from normal fetal frontal lobe (FL27) revealed significant differences [139]. 

The screening experiments conducted under identical instrumental and solution conditions highlighted the incidence of 25 distinct GG species in the An28 mixture and no less than 44 (of which 4 asialylated) in the FL27 mixture. Because of the relatively low resolution exhibited by HCT MS, only 50 different glycoforms could be discriminated altogether. However, the results of the chip ESI HCT MS approach revealed that the individual high-performance TLC (HPTLC) bands reported in the previous study [1] contained distinct GG species with similar migration properties. Interestingly, GD3(d18:1/18:0), GD2(d18:1/18:0), GM1(d18:1/18:0), and their neolacto or lacto-series isomers were detected as ions as singly and/or doubly charged ions of similar low abundances in both mixtures, whilst GT1(d18:1/18:0) and GD1(d18:1/18:0) were found highly expressed in An28. Although both samples were found dominated by mono-, di-, and trisialotetraoses, several glycoforms such as GT1, GQ1, and GQ2 were only/higher expressed in An28. Consequently, the elevated expression of polysialilated GGs, specific for incipient developmental stages, could represent a marker of brain development stagnation that occurs in anencephaly.The present approach also documented the postulated data, according to which GT1b is one of the disease markers, by conducting multistage MS fragmentation experiments. Hence, the MS^2^–MS^4^ analyses on GT1(d18:1/18:0) species detected in An28 sample at *m/z* 1063.72 evidenced the localization of the disialo (Neu5Ac_2_) element at internal galactose moiety and so have disclosed for the first time the presence of GT1b species in the cerebral remnant of the anencephalic brain [1]. 

By employing the same method, a difference in the expression of GGs extracted from of a histopatologically defined anencephalic fetal brain remnant in the 36th gestational week and normal fetal brain was also identified [140]. In this study, GM1a and GD1a were less expressed in An36 compared with the normal fetal brain, whereas only traces of GM1b, nLM1, and nLD1 were found in An36 [140].

Based on the earlier data, we have developed recently the first systematic and comparative MS mapping assay based on Orbitrap MS and MS^n^ for investigating three different GG samples extracted and purified from glial islands of anencephalic residual brain tissue in the 29th(An29), 35th(An35), and 37th(An37) gestational week [138]. This superior approach characterized by ultrahigh sensitivity, resolution, and mass accuracy allowed not only the detection of low-abundant species, such as polysialylated GGs, but also enabled for the first time the detection and identification of no less than 157 distinct components, of which 104 were in An29, 116 were in An35, and 107 were in An37, many of them modified by fucosylation and acetylation or identified de novo.

**Figure 1:**
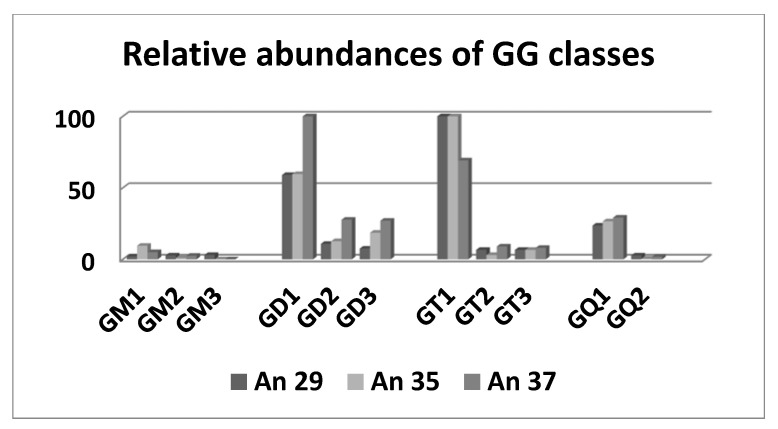
Histogram plotting the changes in the relative abundances of GG classes in An29, An35, and An37 (reprinted with permission from [138]).

**Figure 2:**
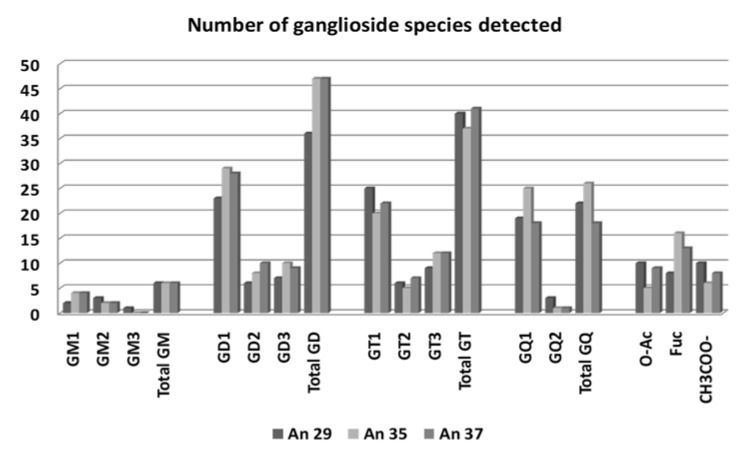
Histogram plotting the number of the identified species in An29, An35, and An37, according to the composition of their glycan core (reprinted with permission from [138]).

Importantly, the overexpression of highly polysialylated GGs in the last trimester of pregnancy, together with the reduced concentration of GM1, as revealed by the histogram in Figure 1 and Figure 2, represent markers of brain development stagnation specific for anencephaly. Additionally, the alteration of the sialylation degree within the three investigated samples and the sensitivity and accuracy of Orbitrap MS revealed the incidence of several GG structures bearing different types of attachment, such as *O*-fucosylation, *O*-acetylation, or (CH_3_COO^−^) modification. While the fucosylated GGs were detected as being overexpressed in An35 and An37, the number of acetylated and modified by (CH_3_COO^−^) GG species was found superior in An29 and An37 [138]. 

Considering that in all reports [1,138,139,140] the polysialylated species were found associated with the reminiscent brain tissue of anencephalic fetuses and could be used as biomarkers, an exhaustive structural characterization of GT2(d18:1/16:2) detected in all three samples was further conducted by CID MS^2^-MS^3^ experiments [138]. Hence, this study not only postulates a new set of GG biomarkers for anencephaly together with a high-performance bioanalytical method optimized for their detection and characterization but also opens new perspectives in early diagnosis through the screening of a mother’s blood for detection of possible anencephaly-related GG species in the first trimester of pregnancy.

## 4. Primary Brain Tumors

Primary brain tumors represent those tumors originating from brain tissue or the brain’s immediate surroundings. The World Health Organization (WHO) classifies primary brain tumors based on cellular origin, histopathologic appearance, and immunohistochemical data. Using a combination of morphological features, growth patterns, and molecular profile, the primary brain tumors are assigned to a certain malignancy grade [141]. While neuroglial tumors, originating from astrocytes, oligodendrocytes, or ependymal cells, represent more than 80% of primary brain tumors, meningiomas developed from meningothelial cells comprise about 20% [142]. The source of primary brain tumors still remains unknown; however, high-dose ionizing radiation andsome inherited disorders including neurofibromatosis, Von Hippel–Lindau syndrome, Li-Fraumeni syndrome, and Turcot syndrome may increase the risk of brain tumors [141,143]. 

A series of representative symptoms including persistent headache, generalized seizures, unilateral weakness nausea, expressive language disorder, vomiting, neurocognitive symptoms, visual problems, confusion, and personality changes were reported by patients with primary brain tumors. The standard approach for diagnosis and treatment of brain tumors includes computed tomography (CT) and magnetic resonance imaging (MRI), followed by surgery, radiation, and/or chemotherapy, although these methods are often challenging due to collateral brain tissue sensitivity to disruption and the toxicity accompanied by side effects of the therapeutic agents. 

Since frequently the symptoms appear in advanced stages, when surgical interventions are often ineffective, in recent years considerable efforts have been invested in identifying possible molecular markers that could be used for an early diagnosis. While immunocytochemistry, flow cytometry, Raman spectroscopy, and Western blotting provide information only on the most abundant glycoforms, and while many less abundant species with a potential biomarker role remain undetected [144,145,146], MS demonstrated its capability to detect and characterize low abundant species in complex mixtures. Hence, the next section describes the achievements in the comprehensive screening and structural characterization of several primary brain tumor glycoconjugate’s composition based on ultrasensitive high-resolution MS.

### 4.1. Meningioma

Meningioma, the most common type of primary brain tumor, is an extra-axial tumor that arises from the meninges, the membranes surrounding the brain and spinal cord [147]. Though not precisely a brain tumor, it is integrated in this group because it may compress or squeeze the adjacent brain, nerves, and vessels, or through CSF, it can invade the surrounding areas, including nearby bone tissue, or it can spread to other organs in the body. Meningiomas are usually diagnosed easily by brain imaging techniques; ninety percent of them are benign, with a low rate of recurrence following surgical resection. Depending on the location, if the tumor is not superficial on the dural surface and accessible, complete removal may be difficult and require post-surgery radiation therapy to avoid recurrence [148], which increases morbidity and even mortality [149,150]. 

Considering this feature, as well as the inconsistency between the biological behavior and tumor grade, novel research directions in bioanalytics and molecular medicine emerged [151] in an effort to discover molecular markers for more untimely tumor detection [152]. After more than five decades since the first assessment of GG composition in human meningiomas by immunochemical and immunohistochemical analyses, the published dataare limited and sometimes controversial. Whereas Kostic and Bucheit [153] revealed a lower GG expression in human meningiomas as compared with the intracranial tumors, Sunder-Plassman and Bernheimer [154] and Aruna et al. [155] indicated an increased GM3 content. However, in the latter study, GM3 accounted for only 15%, whereas GD1a accounted for 33% of the total NeuAc [155]. 

Other data are still consistent with the GM3 elevated content in human meningioma but also report a significantly higher amount of other GG classes, such as of GM1 [156] or GD3 [157,158]. By employing laser densitometric analysis, Radić et al. [159] reported in 2008 differences in serum GG content and composition before and after surgical removal of various brain tumors. Hence, for meningioma, the authors suggested that the postoperative decrease in GD3 proportion and the increase in GM3 proportion might be further used in clinical medicine to follow the tumor progression, surgical success, and prognosis [159].

**Figure 3:**
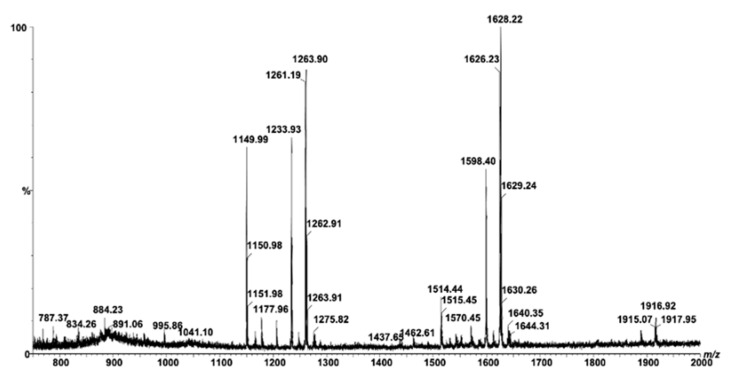
Fully automated (-)nanoESIchip-QTOF MS of the GG mixture from angioblastic meningioma. Sample concentration 2 pmol/L in MeOH; acquisition time 2 min; cone voltage: 30–60 V (reprinted with permission from [152]).

A strategy employing HPTLC and laser densitometry for profiling and quantitative assessment, as well as fully automated chip-based nanoESI (nanoESIchip) on a NanoMate robot in combination with QTOF MS, was developed, optimized, and for the first time applied for mapping and sequencing of GGs expressed in a human meningioma specimen [152]. Hence, the detected meningioma GG content, about 20 times less than the total GG content of healthy human brain tissue determined recently [160], supports the earlier reports [160,161,162] related to the lower GG quantity in brain tumors. While HPTLC profiling revealed only six major fractions, due to the elevated sensitivity, reproducibility, mass accuracy, and ability to discover minor species in complex mixtures, nanoESIchip-QTOF MS highlighted for the first time major changes in the GG profile as compared with the normal brain. The acquired (-)nanoESIchip-QTOF MS is presented in Figure 3, while the detected ions are listed in Table 2. Thirty-four distinct glycosphingolipid components were identified in meningioma, of which five were asialo, one GM4, nine GM3, two GM2, two GD3, nine GM1, and six GD1, differing in their ceramide compositions and bearing no *O*-fucosylation, *O*-acetylation, or *O*-GalNAc modifications, as typical for other brain tumors [152].

Interestingly, the major GG fraction, as expected, belongs to GM3 and accounts for 48.8% of the total GG content, being followed by GD1a (nLD1, LD1) with 34.8%. While the proportion of GD1a (nLD1, LD1) is just slightly above the value corresponding to the healthy brain, GM3 percentage was detected to be over 20 times higher. In addition to the unexpected number of species, another important aspect revealed by MS is the exclusive expression of GGs with shorter glycan chains and low sialic acid content, i.e., maximum sialylation degree found in meningioma is two. Moreover, nanoESIchip MS disclosed that, despite the reduced percentage of GM1 fraction in meningioma, a number of eight abundant ions were associated with nine GM1 forms. Hence, the structural characterization by CID MS/MS focused on GM1 species, previously regarded as species of low relevance for this tumor type, highlighted the incidence of both GM1a and GM1b isomers in meningioma tissue. In view of the current results related to GM1, the authors recommend that further exploration of GM1 role and its consideration as a tumor biomarker, along with GM3, a well-known biomarker of meningioma [152].

### 4.2. Hemangioma

Hemangioma is a congenital benign tumor that features enlarged blood vessels with a single layer of endothelium, together with the lack of neuronal tissue within the lesions [163]. Infantile hemangioma—which appears in the days or weeks after birth and can be localized both on the skin and internal organs, such as brain, liver, kidney, eyes, lungs, bones, spleen, or pancreas—is the most common form of hemangioma [164,165,166,167,168]. On the other side, cavernous hemangiomas, also known as cavernous malformations, cavernous angiomas, or cavernomas, are the most widespread form of benign brain hemangiomas that form during development. This form, characterized by cavities filled with blood with a very thin vascular wall, is specific to the frontal and temporal lobes [169], affects 0.5% of the population of all ages, becomes symptomatic only in 40% of cases, and occurs in the third to sixth decade of life [170]. Most frequently, cavernous hemangioma is accidentally diagnosed during imaging investigations, such as CT scan, MRI, nuclear magnetic resonance (NMR) or ultrasonography (ultrasound) preformed following headaches, convulsions, or neurological deficits reported by the patients [171]. Of extreme importance for the clinical management of hemangioma is its early detection, at an incipient and resectable stage, based on routine screening and cancer biomarker detection prior to the clinical symptoms [163,171]. 

The first assessment of human hemangioma ganglioside expression and structure by MS carried out more than one decade ago provided valuable compositional and structural information, which led to the discovery of several tumor-specific structures [163]. The comparative study of GG distribution in cavernous hemangioma localized in the frontal cortex (HFC) vs. an age- and gender-matched normal frontal cortex tissue (NFC) performed by fully automated chip-based nanoESI HCT MS and CID MS^2^ methodology revealed first a significant difference in the number of GG components, expressed: 29 glycoforms in HFC vs. 43 in NFC. Further evaluation documented the predominance in HFC of species exhibiting short glycan chains characterized by a lower overall sialic acid content:13 of the 29 identified structures were monosialylated species of GM1, GM2, GM3, and GM4-type, 13 disialylated of GD1 and GD2-type, and three trisialylated species of GT1 and GT3-type bearing ceramides of variable constitution. Contrastingly, NFC tissue appeared to contain GG chains not only modified by *O*-Ac and GalNAc attachments but also by a more diverse sialylation: 13 species were found monosialylated (GM), while the rest of 30 polysialylated, bearing up to four Neu5Ac residues (GQ). Interestingly, none of the trisialylated forms detected in HFC were identified in NFC, suggesting that GT1(d18:1/20:0) and GT3(d18:1/25:1) might be associated with HFC. The structural analysis by CID in MS^2^ mode of the low intensity precursor ions corresponding to GT1(d18:1/20:0) and GD1(d18:1/20:0) provided data supporting the presence of all four GT1a, GT1b, GT1c, and GT1d isomers in brain hemangioma tumor, with emphasis on the incidence of an unusual GT1c through the majority of the fragment ions [163].

**Figure 4:**
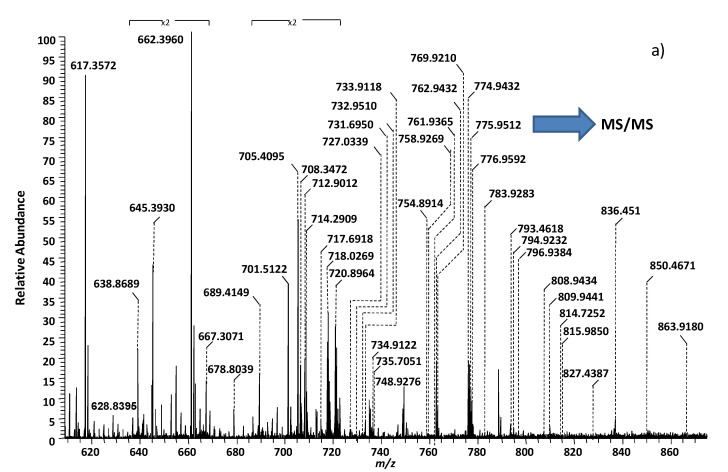

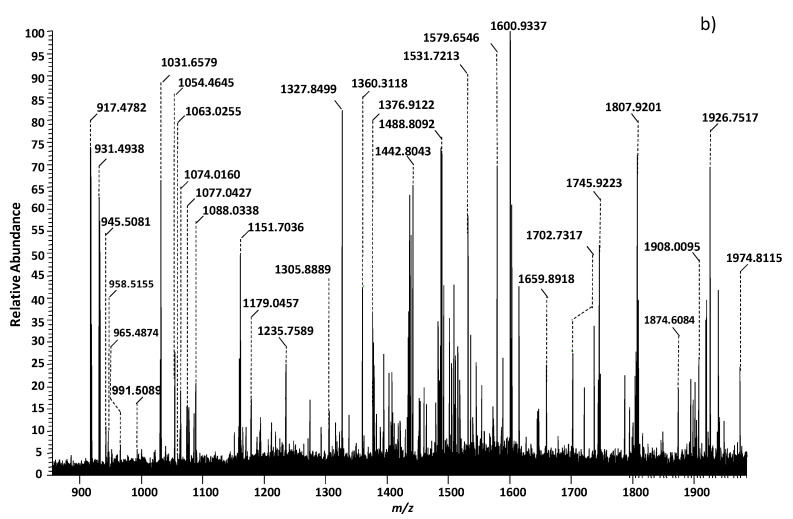
(-)NanoESI Orbitrap MS of HE42 mixture. (**a**) *m*/*z* range (600–900); (**b**) *m*/*z* range (900–2000). Solvent: methanol; sample concentration: 5 pmol/μL; acquisition time: 2 min; spray voltage: 0.65 kV (reprinted with permission from [171]).

A recent revisit of human brain hemangioma (HE42) GGs using an approach based on high-resolution MS on an Orbitrap MS with nanoESI tuned for detection in the negative ion mode [171] revealed a highly complex molecular ion pattern, encompassing species characterized by a prominent diversity of both glycan chains and ceramide moiety compositions, as well as various biologically significant modifications, such as *O*-acetylation, *O*-fucosylation, and CH_3_COO^−^ attachments (Figure 4). 

As visible in Table 3, the high resolution of the instrument allowed the discrimination, identification, and correlation with human hemangioma of over 50 distinct GGs in the mixture, almost double the number of species previously reported as being associated with this tumor.

Of these, 14 were monosialylated, 23 disialylated, 13 trisialylated, and only 1 tetrasialylated and 1 asialylated. The rough quantitative assessment, based on the relative abundances, was disclosed through the higher proportion of GM1, GD1, and GD3 that in cavernous hemangioma mono and disialylated GGs dominated not only numerically but also quantitatively. Moreover, the increased incidence of glycoforms characterized by a low degree of sialylation and short glycan chains, are in agreement with previously achieved data [163] related to the reduced overall sialylation in hemangioma. In view of the elevated incidence of GD3 glycoforms in HE42, the structural analysis by CID MS/MS provided data able to confirm the GD3(d18:1/24:1) and GD3(d18:1/24:2) structures, which might have a potential biomarker role and could be used in early diagnosis [171].

### 4.3. Astrocytoma

Astrocytomas (AcTs), the most common gliomas, are tumors that originate in astrocytes, a type of glial cells star-shaped and localized in the cerebrum [172]. In adult individuals, AcTs are additionally the most common brain tumors, the most aggressive primary brain tumors, and those with the poorest vital prognosis [173]. 

According to the WHO classification based on the growth rate and prospective for proliferation in the adjacent brain tissue, AcTs are divided in four subtypes: (i) pilocytic astrocytoma (grade I), a slow-growing AcT, localized most often in the cerebellum, that usually does not invade into the surrounding brain, and hence, can be completely resected; (ii) diffuse astrocytoma (grade II), a relatively slow-growing invasive tumor, impossible to fully separate from the surrounding brain during surgery, which tends to reappear after treatment; (iii) anaplastic astrocytoma (grade III) characterized by more aggressive features, including a higher growing rate and a larger brain invasion, for which radiation and chemotherapy are required following surgery; and (iv) *glioblastoma* multiforme (grade IV), the most malignant, aggressive, invasive, and common (60%) type of AcT.

Located in nearly all parts of the brain, sometimes even in the spinal cord, AcTs can determine compression, invasion, and destruction of the neural tissue. Typical survival ranges for AcTs are from more than 10 years from the moment of diagnosis for patients with WHO grade I, to about 5 years for WHO grade II, decreasing to 2 to 5 years for WHO grade III, and less than 1 year for patients with WHO grade IV [174].

Usually, AcTs are detected by diagnostic imaging methods, mostly in a late phase, when the standard treatment as a combination of surgery, chemotherapy (mainly TMZ), and radiotherapy only faintly improves patient survival and the quality of life. Hence, new diagnostic methods for early detection of AcTs based on biomarker discovery and arising prior clinical symptoms have been considered lately. The current investigation is focused on the development of proficient analytical methods able to detect molecular fingerprints, among which are the GGs, which were already demonstrated as potential brain cancer biomarkers.

**Figure 5:**
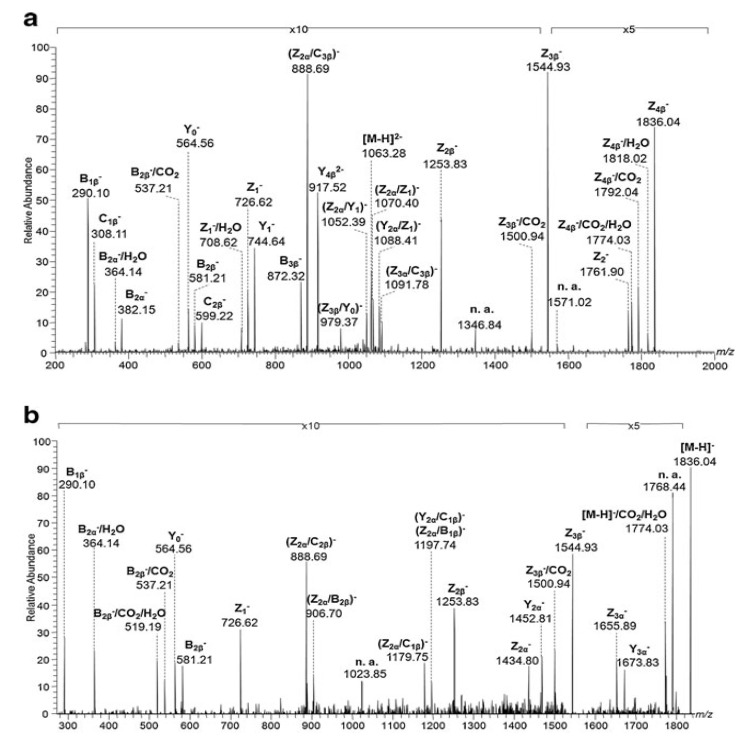

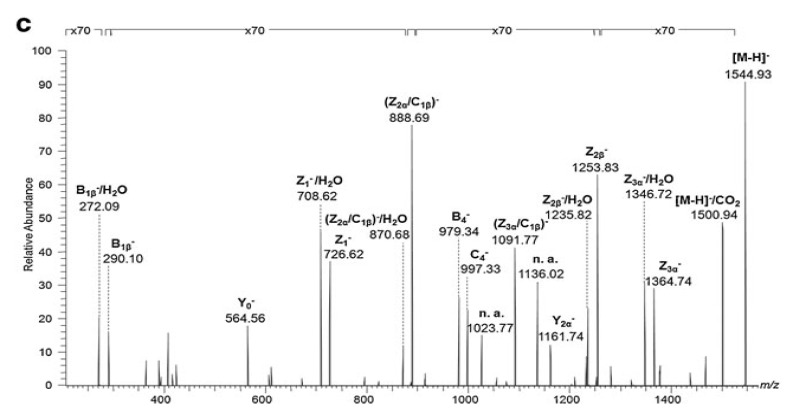
Structural analysis by multistage CID of the [M-2H]^2−^corresponding to GT1(d18:1/18:0) or GT1(d18:0/18:1) detected at *m/z* 1063.28 by MS screening of AcT gangliosides: (**a**) CID MS^2^; (**b**) CID MS^3^ using as the precursor the [M-H]^−^ at *m/z* 1836.04 detected as the Y2^-^ ion in CID MS^2^; (**c**) CID MS^4^ using as the precursor the [M-H]^−^ at *m/z* 1544.93 detected as the B^2-^ ion in CID MS^3^; collision energy within 35–50 eV range. Total acquisition time (MS^2^-MS^4^) 4 min; sample volume consumption 600 nL (reprinted with permission from [175]).

A thorough comparative mapping of GG expression in AcT, its surrounding tissue (ST), and a normal control brain tissue (NT) was for the first time approached by high- resolution MS on an Orbitrap instrument to identify and structurally characterize the potential tumor-associated markers [175]. The comparative (-)nanoESI MS screening revealed an altered GG composition in AcT and ST in comparison with the GG expression in NT. Thus, due to the high resolving power and mass accuracy, no less than 37 different species in AcT, 40 in ST, and 56 in NT could be detected and identified. Additionally, essential findings in relation to the sialylation were reported: (i) unlike NT, where GM-type of species prevail, AcT is dominated by structures with high sialylation content—14 disialylated (GD), 11 trisialylated (GT), 6 tetrasialylated (GQ), and 1 pentasialylated (GP) glycoforms were identified in the AcT biopsy; (ii) while in AcT and NT, the number of species with more than two Neu5Ac moieties is 18 and 17, respectively, ST was found to contain no less than 23 polysialo GGs, of which, 16 were GT, 5 GQ, and 2 GP. Based on previous studies [176] reporting that hypersialylation in AcT might confer a growth advantage to AcT cells, the high levels of sialylation discovered in ST are of high relevance, suggesting the infiltration of AcT cells in the surrounding tissue. Additionally, the elevated levels of fucosylation and acetylation, typical for malignant transformation, identified in ST as compared with NT and AcT, documents as well the phenomenon of AcT cells invasion in the adjacent areas [175]. Another feature distinguishing AcT and ST from NT is represented by the length of the fatty acid chains. While in both AcT and ST 14 GG species contained ceramide moieties with over 25 carbon atoms in the composition of the fatty acids, only five such glycoforms were detected in NT. Interestingly, the MS screening data revealed that the three specimens contain only one common GG structure—namely, GT1(d18:1/18:0) or GT1(d18:0/18:1) detected at *m/z* 1063.31. Hence, the further structural investigation by CID multistage (MS^2^-MS^4^) MS was conducted using as the precursor the ions at *m*/*z* 1063.31. Tandem MS disclosed through the diagnostic product ions the incidence in ST and AcT (Figure 5) of GT1c isomer bearing (d18:1/18:0) and/or (d18:0/18:1) ceramide, but not in NT, to which only the isomer b was found associated. Remarkably, through the association of GT1c isomer to AcT tumor and the surrounding tissue, the MS results not only provided valuable structural data but also were able to indicate that AcT cells invaded the surrounding tissue [175].

### 4.4. Glioblastoma Multiforme

Glioblastoma multiforme (GBM), located most often in the cerebral hemispheres, especially in the frontal and temporal lobes of the brain, is the most widespread primary brain tumor in adults, accounting for 45.2% of malignant primary brain and CNS tumors [177]. GBM, characterized by very abnormal-appearing cell and regions of dead tissue, exhibits not only a highly infiltrative growth pattern invading the nearby brain tissue [178] but also a heterogeneous nature, displaying differences in epigenetic regulation and differentiation status [179,180].

While primary GBMs originate directly as a grade IV tumor (90% of cases) and occur in older patients, with a mean age of 62 years, secondary GBMs (10% of cases) are usually located in the frontal lobe, exhibit a malignant progression, evolve from previously existing lower-grade AcT or oligodendroglioma, and occur in younger patients, with a mean age of 45 years. Moreover, secondary GBMs have a smaller degree of necrosis and carry a greater prognosis than primary GBMs [177]. 

GBM, with an incidence of 3.21 per 100,000 population [181], remains an incurable disease that can result in death in six months or less if untreated. Complicated imaging techniques such as CT or MRI can accurately indicate the location of GBM and its diameter, normally within 5–10 cm [182]. Moreover, intraoperative MRI may successfully help in guiding tissue biopsies and tumor removal. However, given the infiltrative feature of GBM and the inability of neural tissue to regenerate, the total surgical resection of GBM tumor cells is not so efficient since it cannot be performed without extracting surrounding normal brain tissue needed for normal neurological function. Additionally, GBM is refractory to radiation therapy and chemotherapy since the brain-blood barrier impedes the effects of different anticancer reagents [183]. In addition to the fact that patients diagnosed with GBM have an extremely poor prognosis−51.6% 1-year survival rate and 7.8% 5-year survival rate [178]—the recurrence in the peritumoral tissue occurs in about 95% of patients [184].

Nowadays, research in the field of GBM management is directed on one side towards the assessment of the molecular mechanisms related to the tumor aggressiveness and the discovery of novel approaches for invasiveness suppression and on the other side towards the discovery of more effective therapeutic schemes. Since GGs are known as tumor-associated antigens [185,186,187], it is mandatory to achieve an accurate mapping of their expression in GBM in order to understand the role of GD3/GD2 in tumor cell proliferation and invasion [183]. Hence, a detailed characterization of GG composition in GBM in comparison with peritumoral tissue and normal brain tissue, especially addressing the ceramide variability and *O*-acetylation of tumor-associated GGs, was conducted using MS and HPTLC to reveal their roles as tumor-associated antigens [188]. GG expression was investigated in the peritumoral tissue since the biochemical processes within this area are responsible for the aggressive infiltration of tumor cells into the surrounding brain tissue, its recurrence, and poor prognosis [188,189].

The study of Fabris et al. [188] clearly demonstrated distinctive changes in GG expression, showing five times lower total GG content and higher abundance of simple GG structures in GBM. Another important feature is related to the ceramide composition; if GBM GGs were found characterized by highly diverse ceramide compositions, with fatty acyl chains of 16 to 24 carbon atoms, the normal and peritumoral tissue contained mostly C18 chains. Hence, GGs with (d18:1/18:0) and (d20:1/18:0) ceramides were the most abundant in peritumoral and healthy brain tissue, while ceramides with shorter (C16) and longer (C22, C24) fatty acids and unsaturated fatty acids (C24:1), as well as fatty acids with an odd number of carbon atoms (C17, C19), were found to predominate. The peritumoral tissue expressed an elevated abundance of GD3 and nLM1/GM1 species and a lower abundance of GT1 species vs. normal brain. *O*-Ac-GD3 and GD3(d18:1/24:0) were detected exclusively in GBM tissue. Fragmentation analysis of *O*-Ac-GD3(d18:1/18:0) and *O*-Ac-GD3(d20:1/18:0) in CID MS/MS experiments provided clear-cut structural evidence of the novel GBM associated *O*-Ac-GD3 isomer possessing the *O*-acetylation in the inner sialic acid residue, an isomer previously associated with gliomas.

**Figure 6:**
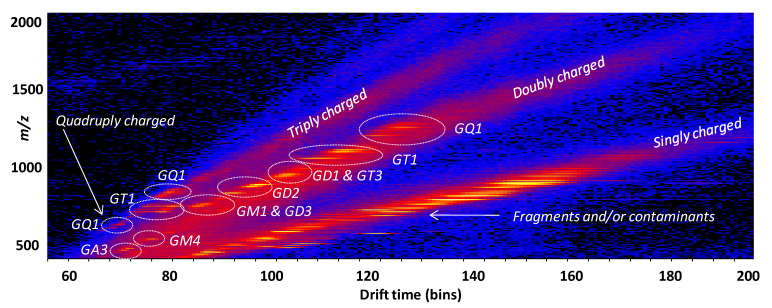
Driftscope display (drift time vs. *m*/*z*) of the total distribution of GG ions from GBM. In the drift cell, GG ions were separated based on the charge state, the carbohydrate chain length, the degree of sialylation, and ceramide composition (reprinted with permission from [183]).

**Figure 7:**
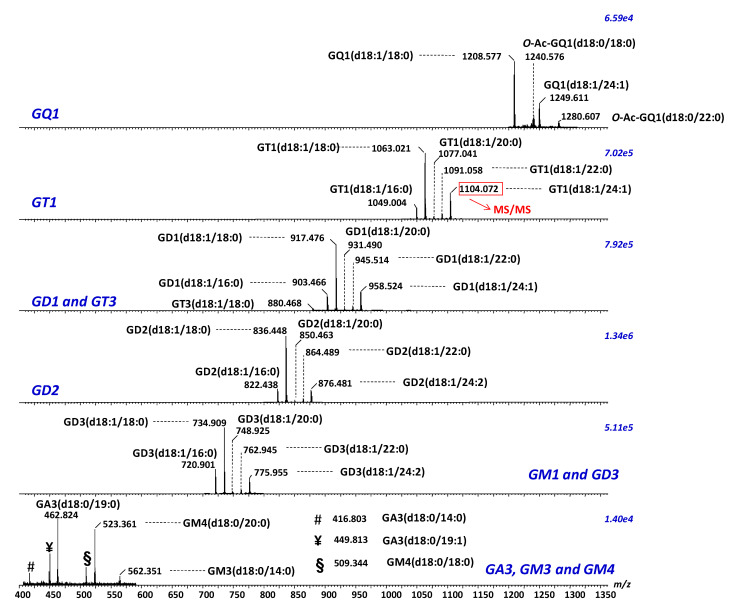
Extracted (-)nanoESI IMS mass spectra of doubly charged GA3, GM3 and GM4; GM1 and GD3; GD2; GD1 and GT3; GT1 and GQ1 from the corresponding areas indicated in Figure 6 (reprinted with permission from [183]).

**Figure 8:**
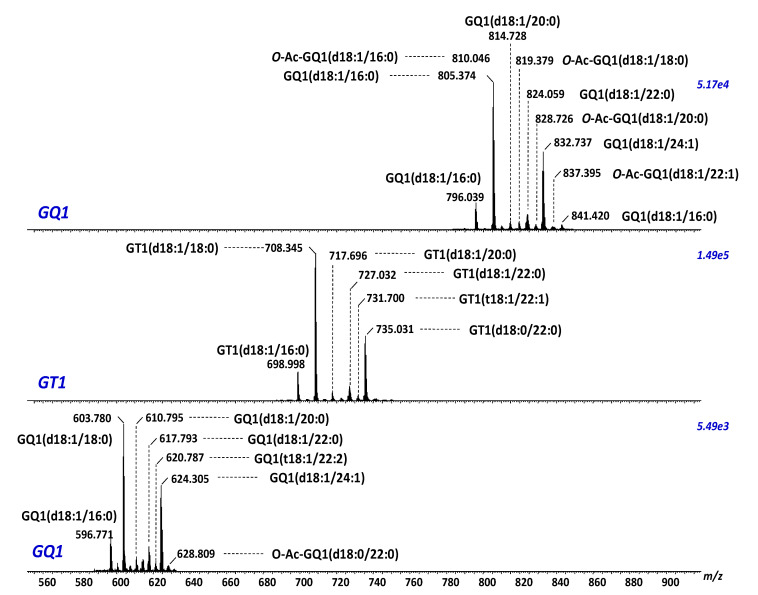
Extracted (-)nanoESI IMS mass spectra of triply charged GT1 (middle), GQ1 (up), and quadruply charged GQ1 (down) from the corresponding areas indicated in Figure 6 (reprinted with permission from [183]).

In view of the poor prognosis of GBM, the need for development of novel and advanced methods capable for an early GBM diagnosis through molecular markers, and the superior potential of ion mobility separation (IMS) MS to discriminate and identify glycoforms in highly complex mixture [190,191,192,193,194], in 2021 IMS MS was introduced for the discovery of GBM-specific structures [183]. A native GG mixture extracted from a brain tumor localized in the frontotemporal cortex of the right hemisphere, clinically diagnosed as GBM using CT and MRI, was analyzed by nanoESI IMS MS in negative ion mode. After only 2 min of signal acquisition, the 2D data set of GBM GGs presented in Figure 6 was obtained. A closer inspection of the DriftScope illustrated in Figure 6 revealed, in addition to the clear distribution of the chemical noise across a wide range of drift times, the GG separation into mobility families based on their charge state, carbohydrate chain length, and the degree of sialylation [183]. The main advantage of IMS MS analyses is the possibility of exporting from DriftScope only the data for regions of interest. Hence, following drift time retention of each narrow area indicated in Figure 6, by combining the scans of the extracted ion chromatograms (XICs), the MS for each GG class was generated, as presented in Figure 7 and Figure 8.

IMS MS offered a reliable separation, given the detection and identification in GBM of 215 ions, corresponding to no less than 160 distinct glycoforms, more than triple the number of GGs previously discriminated in GBM with no separation prior to MS [188]. The inspection of the data has shown that GD3 species predominate in GBM, with 36% of the total number of discovered GGs. Moreover, this study has demonstrated that, in addition to GD3, GT1 species are also associated with GBM [183].

The role of GD3 in brain development is well known; its elevated expression is directly connected with tumor cell proliferation and the invasion and the degree of malignancy. On the other side, GT1b isomer has been previously proposed by Hamasaki et al. [195] as a brain metastasis-associated glycoform, even if never related with GBM. 

In this context, nanoESI IMS CID MS/MS performed in the transfer cell, after ion mobility separation for a GT1 species exhibiting an unusual ceramide composition—namely, (d18:1/24:1) detected at *m/z* 1104.072—has documented, together with the occurrence of a single mobility feature, the presence of the GT1c isomer in GBM. Additionally, the IMS MS approach confirmed the earlier reports related to the high variability of GG fatty acid patterns in human GBM [188] and gliosarcoma [161], a variant of GBM. No less than 50 of the 160 glycoforms bearing ceramides with fatty acyl chains within 22 to 28 carbon atoms were discovered by IMS MS [183].

On the one hand, the major outcome of these studies is that a variety of novel species could be detected, assigned, and added to the currently existing panel of GBM tissue-associated structures, and on the other hand, CID MS/MS led to the discovery of new potential biomarkers, which might be further used in clinical applications [183,188].

### 4.5. Gliosarcoma

Gliosarcoma (GS) is a primary tumor of the CNS characterized by a biphasic growth pattern consisting of malignant glial and sarcomatous components and mesenchymal differentiation often resembling fibrosarcoma [196]. Actually, GS is described as a rare histopathologic variant of GBM, with a slightly greater tendency for temporal lobe involvement. Since it accounts for 2% up to 8% of all GBMs [196,197], gliosarcoma represents an extremely rare neoplasm [196,198], with very limited knowledge about it. 

Despite its rarity, several attempts by Meis et al. [198], Galanis et al. [199], Kozak et al. [200], Frandsen et al. [201], and others [196,197] to characterize GS occurring in adult brain were reported. 

There are several major findings of all studies on GS vs. GBM: (i) GS has a propensity to occur in the elderly, within the fifth to seventh decade of life, being rarely seen in children; (ii) GS shows a slight male predominance (male/female, 1.8) [202]; (iii) GBMs are likely to receive less aggressive surgical resection, with almost a quarter undergoing non cancer-directed surgery compared with less than 10% of GS patients [200]; (iv) just like GBM, GS is also divided into primary GS, if the tumor occurs de novo, without any earlier GBM diagnosis, and secondary GS, if the tumor occurs after treatment of conventional GBM [201]; (v) the same treatment as for GBM should be applied for patients with GS; (vi) after surgical resection, chemotherapy, and radiotherapy, the prognosis remains very poor, being slightly worse than that observed for GBM patients. The median survival among all GS patients was 9 months.

**Figure 9:**
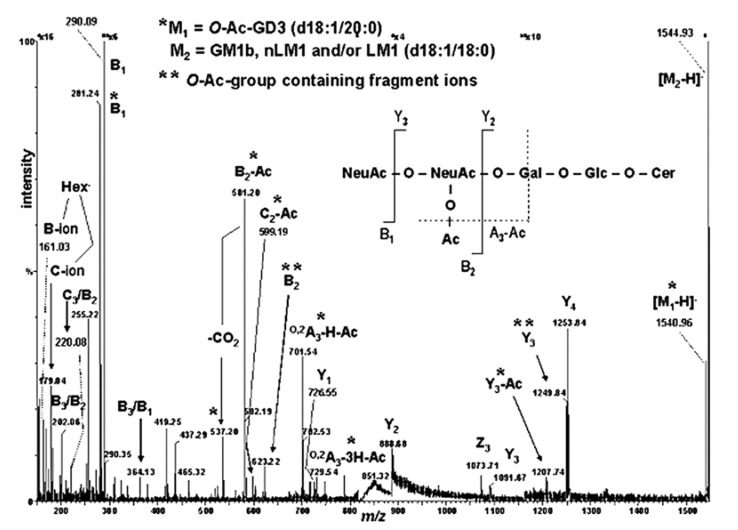
Negative ion mode nanoESI QTOF MS/MS of the [M-H]^2-^ ion at *m/z* 1540.96 corresponding to the *O*-Ac-GD3 (d18:1/20:0). ESI voltage, 1000–1250 V; for precursor ions isolation the LM and HM parameters were set to 3; collision energy: 25–40 eV; collision gas pressure: 5–10 psi.; acquisition time, 11 min; average sample consumption, 3.5 pmol. Inset, the fragmentation scheme of *O*-Ac-GD3 (reprinted with permission from [161]).

The tumor aggressiveness, which causes extensive infiltration of the tumoral cells into the surrounding healthy brain tissue, is responsible for the failure of GS treatment methods. A treatment strategy considered already for several years is to target the invading tumor cells by using specific binding ligands [203]; however, the critical point is the identification and thorough characterization of the tumor-specific target molecules. Given the role of GGs in malignant transformation and tumor progression/invasiveness, an ample project focused on the mapping of GS biomarkers vs. normal brain tissue, by high-resolution MS using chip-based nanoESI QTOF and ESI Fourier-transform ion cyclotron resonance (FTICR) MS for screening and CID for structural elucidation was undertaken [161]. The combined MS approach revealed a highly altered GG expression in GS as compared with the normal brain tissue. The applied MS strategy enabled the detection and structural assignment of 73 distinct species in GS, many more than ever reported for investigated gliomas. In addition to the 7.4 times lower total GG content in GS vs. normal human brain, the tumor was found to contain all major brain-associated species, though in much altered proportions. Hence, the highly distinctive GD3 (48.9%) > GD1a/nLD1 > GD2/GT3 > GM3 > GT1b > GM2 > GM1a/GM1b/nLM1 > LM1 > GD1b > GQ1b pattern was allied with GS. Among the GD3 class, the species bearing (d18:1/18:0), (d18:1/24:1), and (d18:1/24:0) ceramides were found dominant in GS. Surprisingly, a high abundance of counterpart *O*-acetylated GD3 derivatives, particularly (d18:1/18:0) and (d18:1/20:0), was also observed. While GM2, GM1, and/or their isomers nLM1 and LM1 and GD1 species exhibiting elevated heterogeneity in the composition of their ceramide moieties were found over expressed in the mixture, glycoforms with a higher sialylation degree, such as GT1 were poorly or not at all expressed. *O*-Ac-GM3 (d18:1/20:0), (d18:1/22:1), (d18:1/22:0), (d18:0/22:0), and (d18:1/24:2); di-*O*-Ac-GD3(d18:1/20:0); and *O*-Ac-GT3(d18:1/24:1) and (d18:1/24:0) were also identified in GS by MS as minor ions. CID MS/MS analyses were further used to validate the presence of *O*-Ac-GD3(d18:1/20:0) detected as [M-H]^−^ at *m/z* 1540.88. The product ion spectrum illustrated in Figure 9 documents for the first time the incidence of a GS-associated isomer bearing *O*-acetylation at the inner Neu5Ac residue [161]. 

Responsible for both the reduction in the total GG content and the altered pattern in GS vs. control tissue are considered to be the lower overall biosynthetic rate, caused by changes in expression of certain glycosyltransferases and the higher turnover rate [161]. The most reasonable causes of the elevated GD3 and reduced GM1a, GD1a, and GD1b abundances in GS are the higher expression of sialyltransferase II (GD3 synthase) and the lower expression of galactosyltransferase II [161].

The MS results have shown for the first time that GS contains a higher amount of potentially proapoptotic GD3 than *O*-acetyl GD3 glycoforms. At the same time, MS validated the earlier hypothesis [204], according to which the role of *O*-Ac-GD3 as a tumor-specific element is for the protection of tumor cells from apoptosis. 

## 5. Secondary Brain Tumors

The development of an aberrant cell surface–glycocalyx–molecular composition is a typical process observed in the malignant transformation. This event is followed by the overexpression of certain molecular components, mostly sialylated, which are associated with the potential of cancer cells to metastasize and invade the surrounding areas. As discussed above, GGs are among the molecules carrying glycosyl epitopes able to cause such effects; since GGs regulate cell adhesion/motility, they are prone to initiate tumor metastasis. Consequently, many tumor-associated GG species were found to induce invasive and metastatic phenotypes of tumor cells [32]. In the case of neuroectodermal neoplasms, several studies have shown that the overexpression of b series GGs promotes tumor invasion and aggressiveness [32,205]. On the other hand, GM3, GD3, and GD2 species were postulated as associated antigens, which play a key role in tumor development in the case of melanoma [190] and neuroblastoma [206], which are also neuroectoderm-derived cancers. As a result, disialogangliosides in particular started to attract interest as target molecules for immunotherapy oriented against such forms of cancer [32,205,207].

As compared with other malignant tumors, which spread to the brain only in the most advanced stages of the disease, lung adenocarcinoma (LADC) develops relatively early brain metastases as a major disease complication [208]. Almost 10% of the patients have brain metastases already at the time of LADC diagnosis, whereas up to 50% of patients will develop them during the course of the disease. LADC is the most widespread type of lung cancer (LC), accounting for about 30 percent of all LCs and half of all non-small cell LCs, the later form being the most common [209]. Typically, LADC grows in the outer region of the lungs, most often in the alveoli. As a histologically distinct form of LC, LADC tumor tissue is characterized by well-defined malignant elements as well as cytological and molecular features of high specificity [210].

Left untreated, LADC-derived brain metastases cause death in a few weeks, with almost all patients succumbing because of neurological rather than systemic issues. Surgical resection, stereotactic radiosurgery (SRS), and the gold standard—that is, whole-brain radiation therapy (WBRT)—are currently the primary treatment options for patients suffering from LADC-derived brain metastasis [211,212], although even under treatment, the prognosis is generally poor, with a median survival time between 3 and 6 months.

From the molecular point of view, non-small cell LC is rather characterized by an overexpression of GD3 and 9-*O*-acetyl GD3, GM3, and GM3 synthase (sialyltransferase-I or SAT-I) mRNA with a positive correlation between expression levels of SAT-I mRNA and GM3 in tumor tissues. Another GG of interest in the case of LC and used in development of novel therapies is fucosyl-GM1, found specifically expressed in LC cells [213]. 

**Figure 10:**
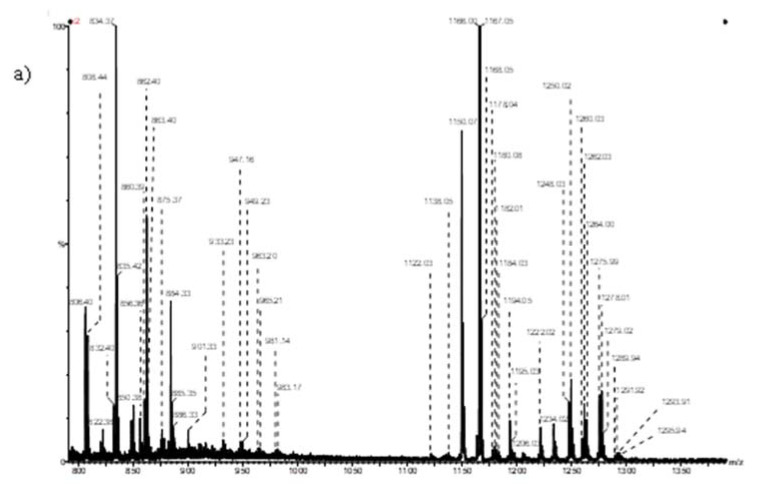

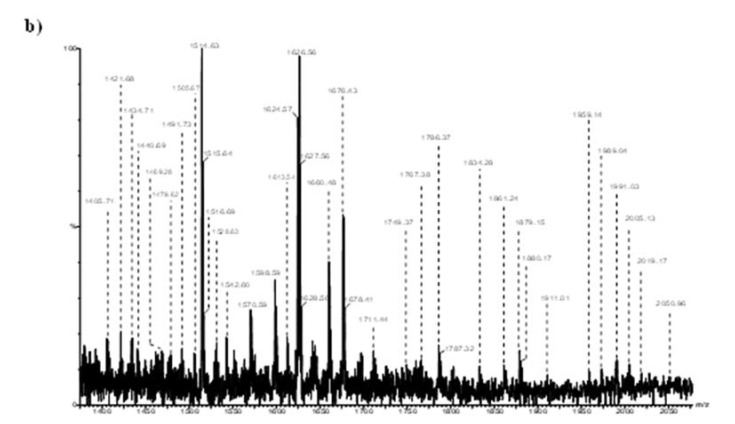
(-) Chip-nanoESI QTOF MS of the native GG mixture isolated from brain metastasis of LADC. Solvent: MeOH; sample concentration 2.5 pmol/μL; acquisition time 1 min; Chip ESI: 1.5 kV; Cone voltage: 45 V. Zoomed area: (**a**) *m*/*z* (800–1350); (**b**) *m*/*z* (1400–2100) (adapted with permission from [160]).

In view of these findings, which connect certain GG species with LADC, the MS-based research in the field was oriented towards the optimization and application of chip-based nanoESI for the investigation of GG expression in LADC-derived brain metastasis and discovery of biomarkers that might be useful in diagnosis and therapy. In a report focused on biomarkers of LADC brain metastasis [160], a native GG mixture extracted and purified from a tissue specimen of LADC brain metastasis (male patient, 73years old) was screened by a NanoMate robot coupled to a QTOF MS (Figure 10a,b) vs. a control sample—a native GG mixture extracted from age-matched healthy brain tissue, sampled, purified, and analyzed under identical conditions. The comparative assay highlighted a considerable difference in the number and type of GG components expressed in LADC-brain metastasis (Table 4) vs. normal cerebellar tissue (Table 5).

The control tissue was found to contain a higher variety of structures differing in their sialylation degree—from short, monosialylated (GM) to large, polysialylated carbohydrate chains (GH)—and also GG chains modified by *O*-acetyl (*O*-Ac) and fucosyl (Fuc) attachments. GM1(d18:1/18:0) or (d18:0/18:1), GM1(d18:1/20:0) or (d18:0/20:1), and Fuc-GM1(d18:1/18:0) were detected as abundant singly charged ions at *m/z* 1544.92, 1572.92, and 1690.95, respectively. In addition to these species, highly abundant doubly charged ions at *m/z* 917.44 and 931.45 assigned to disialylated GD1 components, with (d18:1/18:0) or (d18:0/18:1) and (d18:1/20:0) or (d18:0/20:1), respectively, were identified. 

The healthy brain was also found dominated by mono-, di-, and trisialylated structures. A total of 28 distinct *m/z* signals corresponding to 44 possible GM-type species, 44 *m/z* signals corresponding to 63 possible GD-type species, and 32 *m/z* signals attributable to 59 GT-type of species were detected. Most of these structures have a tetrasaccharide sugar core and exhibit high heterogeneity in their ceramide composition. Additionally, six possible tetrasialylated structures (GQ) and only one asialo species (GA) were identified. Notable is the presence of a hexasialylated GH2 having (d18:0/24:1) or (d18:1/24:0) Cer constitution. This glycoform was detected as [M-3H]^3−^ at *m/z* 968.34 in anormal biopsy and was not found in the pathological brain sample. Eighteen possible components modified by fucosylation, as well as 30 possible *O*-acetylated GG variants, were also identified. Most of the fucosylated components are of GM1- and GD1-type with different fatty acid and/or sphingoid base compositions in the Cer moiety. Unlike fucosylation, *O*-acetylation was found as a modification for a high variety of glycoforms, such as GM3, GM1, GD3, GD2, GD1, GT3, GT2, GT1, and GQ1, which differ not only in the oligosaccharide chain composition but also in their sialylation status. Interestingly, four possible di-*O*-Ac variants of GT2, GM1, and GM3 were detected as well. 

In contrast with the healthy cerebellar tissue, the GG mixture extracted from brain metastasis of LADC exhibited mostly species of short oligosaccharide chains and reduced overall sialic acid content. More than a half, from the 59 different ions detected and corresponding to 125 possible structures in the metastatic tissue, represented monosialylated species of GM1-, GM2-, GM3-, and GM4-type. In addition to the large number of monosialylated components, eight asialo species of GA1- and GA2-type bearing ceramides of variable constitution were discovered. GD1, GD2, and GD3 as well as GT1, GT2, and GT3 with short carbohydrate chains, expressing different ceramide portions, were also identified in the mixture. Ganglioside components modified by Fuc or *O*-Ac could also be detected but in a different pattern than in the healthy brain; most *O*-acetylated GGs are monosialo species of GM3, as well as short GT3- and GT2- type, while fucosylated components are represented by monosialo species of GM3 and GM4 structure, di-, and trisialylated GD1 and GT3 exhibiting high heterogeneity in their ceramide motifs.

The most abundant singly charged ions at *m*/*z* 1150.17, 1168.01, 1515.29, and 1627.41 were assigned to GA2(d18:0/22:0) or GM3(d18:1/16:1); GM3(t18:0/16:0); sodiated GD3 (d18:1/18:0) or (d18:0/18:1) or GM1 (d18:1/16:1) or (d18:0/16:2) or GM1 (d18:2/16:0) or *O*-Ac-GD3(d18:0/18:0); and sodiated GD3(d18:1/26:0) or (d18:0/26:1) or GM1(d18:0/24:2) or (d18:1/24:1) or (d18:2/24:0). Hence, the MS data indicated the presence in the metastatic tissue of several unusual monosialylated species modified by fucosylation or *O*-acetylation, such as Fuc-GM4, Fuc-GM3, di-*O-*Ac-GM3, and *O*-Ac-GM3. These species were also reported as fetal brain-associated structures, i.e., developmentally regulated antigens that are only minor components of the normal brain [139]. 

GD3 and GM3 discovered in this study were also shown to enhance tumor cell proliferation, invasion, and metastasis in a variety of brain tumor cells, especially in glioma [161] and neuroblastoma, respectively [206]. GD3 influences tumor angiogenesis and metastasis by stimulating VEGF release from tumor cells, hence its structural characterization is of high biological importance. By tandem MS using CID, the oligosaccharide core of the LACD brain metastasis-associated GD3(d18:1/18:0) species was structurally elucidated in this work. At the same time, a number of Cer-derived fragment ions also allowed the postulation of the lipid moiety composition. 

Optimized MS/MS conditions also enabled the structural assessment of Fuc-GM1(d18:1/18:0) detected in the healthy brain. It was found that the identified Fuc-GM1 is an atypical isomer bearing the labile Fuc residue at the inner Gal molecule, together with one Neu5Ac attached at the same monosaccharide. 

Finally, it is worth highlighting that chip-nanoESI QTOF MS and CID MS/MS were able to provide compositional and structural characterization of native GG mixtures from secondary brain tumors with a remarkable analysis pace and sensitivity, as well as the postulation of novel GG biomarkers. Technically, in view of the flow rate delivered by the chip-nanoESI, which under the applied conditions was around 100 nL/min, 1 min acquisition time at a sample concentration of only 2.5 pmol/μL corresponded to 250 fmol biological extract consumption. Thus, the MS screening followed by CID MS/MS required only 500 fmols of material. For these reasons, over the past few years, this bioanalytical strategy demonstrated for the determination of glycosphingolipid markers of brain tumors could be developed into an efficient tool, largely applied to glycomics of human nervous system [214].

## 6. Conclusions

Gangliosides represent one of the most important molecular pillars of the CNS, whether expressed in health or terrifying diseases. To achieve novel insights into their functional characteristics, understand the many biological events in which these molecules are implicated, and exploit them accordingly as markers of brain diseases or therapeutic agents, the scientists in the field rely on the advancements in bioanalytics and biotechnology. In the post-genome era, MS has developed continuously as one of the most powerful and sensitive analytical techniques for structural elucidation of individual compounds, molecular aggregates, or raw mixtures originating from biological matrices. As inferable from this review, obviously the MS potential in the study of human brain gangliosidome and disease biomarker discovery has increased after the introduction of nanoESI and MALDI methods, the development of high-resolution instruments, microfluidics, and chip-based and/or robotic systems, as well as efficient dissociation in tandem or multistage MS experiments. The latter method gives the possibility of fragmenting structurally complex ionic species in order to acquire information at a deep molecular level. Another recent breakthrough is represented by the optimization and introduction in human brain GG research of IMS MS, a technique able to decipher GG isomers, isobars, and conformers, as well as to provide data on the stoichiometry, topology, and species with biomarker value. In combination with a fragmentation method, such as CID, IMS MS has proven to be an outstanding platform, capable of separation and characterization of even minor compounds in brain GG extracts in a single run and on a single instrument.

The clinical applications of all these systems, which represent the state-of-the-art in MS development, demonstrate not only their performance but also their high versatility in the discovery of biomarkers related to a broad spectrum of brain ailments. By employing such MS-based methods, the gangliosidome associated with neurodegenerative and neurodevelopmental disorders and primary benign and malignant tumors, as well as brain metastases, could be approached and elucidated in detail, most often in demanding comparative assays that require ultrahigh reproducibility and data accuracy. The modern MS approaches discussed here have also shown their potential in establishing well-defined sets of GG fingerprints that are valuable in early diagnosis and assessment of disease evolution or as target molecules for implementation of more efficient therapeutic schemes. 

In view of the rapid developments in the field, we believe that in the near future MS based on fast, flexible, user-friendly, and integrated platforms will be able to cut across some of the traditional methodologies for diagnosis of brain diseases. Moreover, it is expected that innovative microfluidics and IMS MS protocols with a much broader applicability in glycosphingolipidomics will be conceived. 

## Figures and Tables

**Table 1:** Classification and characterization of cephalic disorders.

	Disease	OMIM/Phenotype MIM Number	Mutated Gene or Pathway/Locus	Characterization	Symptoms	Prognosis	Incidence
More common cephalic disorders	Anencephaly	206500 619452	NUAK12 NUAK2	failure of neural tube closure at the cephalic end	blindness, deafness, unconscious, unable to feel pain	death before birth, although some may survive a few hours or a few days after birth [99]	1:1000 pregnancies
	Colpocephaly	-	COL4A1	significant enlargement of the occipital horns	small head circumference, intellectual disability, movement abnormalities, muscle spasms and seizures, poor vision, speech and language difficulties, deafness, and chorioretinitis [100]	depends on the severity of the associated conditions and the degree of abnormal brain development	only 50 cases have been reported up to now
	Holoprosencephaly	236100	HPE1	failure of the prosencephalon to develop	cyclopia, ethmocephaly, anophthalmi, microphthalmia, proboscis, hypotelorism, seizures, hydrocephalus, neural tube defects, pituitary dysfunction, short stature, feeding difficulties, instability of temperature, heart rate, and respiration, developmental delay, and intellectual disability [101]	stillbirth or up to maximum 12 months	1:16,000 live births 1:5–10,000 (Europe)
	Ethmocephaly	-	-	failure of diverticularization and rotation of the prosencephalon into two normal cerebral hemispheres	proboscis situated above hypoteloric orbits in the midline, microphthalmos, absent nasal structures, low-set malformed ears [102]	incompatible with life	1:15,000 live births 1:250 among abortuses
	Hydranencephaly	-	NDE1	the cerebral hemispheres are absent and replaced by sacs filled with cerebrospinal fluid	hypertonia, seizures, hydrocephalus, visual impairment, lack of growth, deafness, blindness, paralysis, and intellectual disabilities [103]	several years or more	1:5–10,000 worldwide
	Iniencephaly	-	-	combines extreme retroflexion of the head with severe defects of the spine	anencephaly, cephalocele, cyclopia, agnathia, cleft palate, arthrogryposis, clubfeet, holoprosencephaly, spina bifida, low-set ears, pulmonary hypoplasia, omphalocele, gastroschisis, cardiovascular disorders, diaphragmatic hernias, gastrointestinal atresia, single umbilical artery, renal abnormalities, genu recurvatum, hydramnios	spontaneous abortion or stillborn	1:5–10,000 (Europe)
	Lissencephaly	607432	PAFAH1B1 DCX, TUBA3 ARX	smooth or nearly smooth cerebral surface due to the lack of normal convolutions in the brain	feeding and swallowing problems, seizures, severe psychomotor retardation and muscle spasticity or hypotonia [104]	up to the age of 10	1:100,000 births
	Megalencephaly	155350 248000	PI3K-AKT	abnormally large, heavy, and usually malfunctioning brain	autism, severe seizures, hemiparesis, and intellectual disability	do not survive through adulthood	1:50 births
	Microcephaly	251200	MCPH1—MCPH12	a much smaller head than normal because the brain has not developed properly	impaired cognitive development, delayed motor functions and speech, facial distortions, dwarfism or short stature, hyperactivity, seizures, difficulties with coordination and balance, feeding difficulties, and other brain or neurological abnormalities [105]	depends on associated medical conditions	2–12:10,000 births
	Porencephaly	175780 614483	COLGALT1 [106]	a cyst or cavity filled with cerebrospinal fluid develops in the brain during fetal development or soon after birth	developmental delay, hypotonia, seizures, macrocephaly or microcephaly, spastic hemiplegia, speech problems, delayed growth, and intellectual disability, epilepsy, hydrocephalus, spastic contractures [107,108]	varies according to the location and extent of the cysts or cavities	1:19,000 in Japan 1:28,500 in the USA
	Schizencephaly	269160	SIX3 SHH EMX2 COL4A1	abnormal slits, or clefts, in the cerebral hemispheres	developmental delays, delays in speech and language skills, and problems with brain–spinal cord communication, abnormally small head, cognitive delay and impairment, partial or complete paralysis, or poor muscle tone, seizures [109]	varies depending on the size of the clefts and the degree of neurological deficit	1:64,935 in USA 1:69,444 in UK
Less common cephalies	Acephaly	-	DCTN5 LHX1 OTX2 [110]	manifests in a twin pregnancy where the head and heart fails to form on one of the embryos, absence of the head	body with no head and heart; the fetus’s neck is attached to the normal twin	not compatible with extrauterine survival	1:34,600 deliveries [111]
	Exencephaly	-	MSX2	partial or complete absence of the calvaria with abnormal development of brain tissue	cranial vault is absent or poorly formed, cephalad to orbits, the nasal bone may be absent in most cases, brain tissue is herniating or dangling in amniotic fluid, brain tissue may be attached to the amniotic membrane [112]	incompatible with extrauterine life	3:10,000 pregnancies [113]
	Otocephaly	202650	PRRX1	mandibular hypoplasia/agnathia, melotia and/or synotia, microstomia with oroglossal hypoplasia/aglossia	holoprosencephaly, skeletal, genitourinary, cardiovascular anomalies, situs inversus, polyhydramnios	incompatible with life	1:70,000 births [114]
	Craniosynostosis	218500	FGFR2 FGFR3 FGFR1 TWIST1 EFNB1 [115]	premature closure of cranial sutures, causing problems with normal brain and skull growth	abnormallyshapedskull/restricted brain growth, increased pressure in the skull, impaired brain development, seizures, blindness and/or intellectual disability, obstructive sleep apnea [116,117]	varies depending on whether single or multiple cranial sutures are involved or other abnormalities are present	1:2100–2500 births
	Brachycephaly	123100 615314 616602	FGFR3 P250R	premature fusion of both coronal sutures leading to skull deformity with a broad flat forehead and palpable coronal ridges [118]	short skull	usually good	1:20,000 births
	Oxycephaly	123100 616602	FGFR2 MEGF8 TWIST1 [119]	a premature closure of the bony vault of the skull	top of the skull is pointed/cone-shaped, blindness, syndactyly and aracluiodactyly, mental deficiency, elevated intracranial pressure [120]	complete recovery after cranial expansion surgery	<1:1,000,000 births
	Plagiocephaly	123100 615314 616602	NIPBL	an asymmetrical distortion of the skull	motor and language developmental delays, head deformities, increased pressure inside the head, seizures, developmental delay [121]	recovery with helmet treatment after surgery [122]	46:100 births [123]
	Scaphocephaly	123100 600775 615529	TWIST1	premature fusion of the sagittal suture	misshapen skull; development of a raised, hard ridge along affected sutures; slow/no growth of the head as the baby grows	recovery after surgery	1:2000 live births [124]
	Trigonocephaly	190440	FGFR1	premature closure of the metopic sutures	delay in language development, hyperactivity, autistic tendencies, and motor dysfunctions, palpable metopic ridge	recovery after surgery	1:700 up to 1:15,000 newborns globally (differs per country) [125,126]

**Table 2:** Proposed composition of single components in the GG mixture from angioblastic meningioma specimen as detected by fully automated (-)nanoESIchip-QTOF MS (reprinted with permission from [152]).

*m/z* (Monoisotopic)	Molecular Ion	Proposed Structure
787.37	[M-H]^−^	LacCer(d18:1/11:2)
834.26	[M-H]^−^	LacCer(d18:1/14:0)
884.32	[M-H]^−^	LacCer(d18:1/18:2)
891.06	[M-H]^−^	LacCer(d18:0/18:0)
995.86	[M-H]^−^	GA2(d18:0/11:0)
1041.10	[M-H]^−^	GM4(d18:1/20:2)
1149.99	[M-H]^−^	GM3(d18:1/16:1)
1151.98	[M-H]^−^	GM3(d18:1/16:0)
1167.70	[M-H]^−^	GM3(t18:1/16:0)
1177.96	[M-H]^−^	GM3(d18:1/18:1)
1233.93	[M-H]^−^	GM3(d18:1/22:1)
1259.90	[M-H]^−^	GM3(d18:1/24:2)
1261.91	[M-H]^−^	GM3(d18:1/24:1)
1275.82	[M-H]^−^	GM3(t18:1/24:2)
1277.80	[M-H]^−^	GM3(t18:1/24:1)
1437.65	[M-H]^−^	GM2(d18:1/22:1)
1462.61	[M-H]^−^	GM2(d18:1/24:2)
1489.55	[M-H]^−^	GD3(t18:0/18:0)
1514.44	[M-H]^−^	GD3(d18:0/21:0) or *O*-Ac-GD3(d18:0/18:0)
1542.41	[M-H]^−^	GM1(d18:1/18:1)
1570.45	[M-H]^−^	GM1(d18:1/20:1)
1598.40	[M-H]^−^	GM1(d18:1/22:1)
1626.23	[M-H]^−^	GM1(d18:1/24:1) or GM1(d18:0/24:2)
1628.22	[M-H]^−^	GM1(d18:1/24:0)
1640.35	[M-H]^−^	GM1(d18:1/25:1)
1644.31	[M-H]^−^	GM1(d18:0/25:0)
1659.32	[M-H]^−^	GM1(t18:1/25:0)
1792.69	[M-H]^−^	GD1(d18:1/15:1)
1833.98	[M-H]^−^	GD1(d18:1/18:1)
1861.95	[M-H]^−^	GD1(d18:0/20:1)
1915.07	[M-H]^−^	GD1(t18:1/24:3)
1916.92	[M-H]^−^	GD1(d18:1/24:2)
1919.86	[M-H]^−^	GD1(d18:1/24:0) or GD1(t18:1/23:1)

d, dihydroxy sphingoid base; t, trihydroxy sphingoid base.

**Table 3:** Assignment of the ions detected by (-)nanoESI Orbitrap MS in HE42 mixture (reprinted with permission from [171]).

Nr. crt.	Proposed Structure	*m/z*_exp_	Molecular Ion
1	GM3(d18:0/24:0)	645.3930	[M-4H^+^+2Na^+^-H_2_O]^2−^
2	GM3(t18:0/24:0)	662.3960	[M-4H^+^+2Na^+^]^2−^
3	GT1(d18:1/18:0)	708.3472	[M-3H^+^]^3−^
4	GD3(d18:0/16:0)	712.9012	[M-2H^+^-H_2_O]^2−^
5	GD3(d18:1/16:0)	720.8964	[M-2H^+^]^2−^
6	GT1(d18:1/20:0)	717.6918	[M-3H^+^]^3−^
7	GT1(d18:1/22:0)	727.0339	[M-3H^+^]^3−^
8	GT1(t18:1/22:1)	731.6950	[M-3H^+^]^3−^
9	GM2(d18:1/24:0)	732.9510	[M-2H^+^]^2−^
10	GD3(d18:1/18:1)	733.9118	[M-2H^+^]^2−^
11	GD3(d18:1/18:0)	734.9122	[M-2H^+^]^2−^
12	*O*-Ac-GT1(d18:0/22:0)	735.7051	[M-3H^+^-H_2_O]^3−^
13	GD3(d18:1/20:0)	748.9276	[M-2H^+^]^2−^
14	GM1(d18:1/14:0)	754.8914	[M-3H^+^ +Na^+^]^2−^
15	GM1(d18:0/16:0)	758.9269	[M-2H^+^]^2−^
16	GD3(d18:1/22:1)	761.9365	[M-2H^+^]^2−^
17	GD3(d18:1/22:0)	762.9432	[M-2H^+^]^2−^
18	GM1(d18:1/18:2)	769.9210	[M-2H^+^]^2−^
19	GD3(d18:1/24:2)	774.9432	[M-2H^+^]^2−^
20	GD3(d18:1/24:1)	775.9512	[M-2H^+^]^2−^
21	GD3(d18:1/24:0)	776.9592	[M-2H^+^]^2−^
22	GM1(d18:0/18:0)	783.9283	[M-3H^+^+Na^+^]^2−^
23	GD2(d18:0/13:0)	793.4618	[M-2H^+^-H_2_O]^2−^
24	GM1(d18:0/18:0)	794.9232	[M-4H^+^+2Na^+^]^2−^
25	GM1(d18:1/20:0)	796.9384	[M-3H^+^+Na^+^]^2−^
26	GM1(d18:0/20:0)	808.9434	[M-4H^+^+2Na^+^]^2−^
27	GM1(d18:0/20:0)	809.9441	[M-4H^+^+2Na^+^]^2−^
28	GQ1(d18:1/20:0)	814.7252	[M-3H^+^]^3−^
29	GD3(d18:1/28:0)	815.9850	[M-3H^+^+Na]^2−^
30	GD2(d18:1/18:0)	827.4387	[M-2H^+^ -H_2_O]^2−^
31	GD2(d18:1/18:0)	836.4515	[M-2H^+^]^2−^
32	GD2(d18:1/20:0)	850.4671	[M-2H^+^]^2−^
33	Fuc-GM1(d18:1/18:3)	863.9180	[M-4H^+^+2Na^+^]^2−^
34	GD1(d18:1/18:0)	917.4782	[M-2H^+^]^2−^
35	GD1(d18:1/20:0)	922.4835	[M-2H^+^-H_2_O]^2−^
36	GT3(d18:1/25:1)	928.5061	[M-2H^+^]^2−^
37	GD1(d18:1/20:0)	931.4938	[M-2H^+^]^2−^
38	GD1(d18:1/22:0)	945.5081	[M-2H^+^]^2−^
39	*O*-Ac-GD1(d18:1/20:1)	951.4899	[M-2H^+^]^2−^
40	GD1(d18:1/24:1)	958.5155	[M-2H^+^]^2−^
41	Fuc-GT3(d18:1/20:2)	965.4874	[M-2H^+^]^2−^
42	Fuc-GT3(d18:1/20:1)	966.4971	[M-2H^+^]^2−^
	*O*-Ac- GD1(d18:1/22:0)	966.4971	[M-2H^+^]^2−^
43	Fuc-GD1(d18:0/18:0)	991.5089	[M-2H^+^]^2−^
44	*O*-Ac-GM4(d18:1/16:0)	1031.6579	[M-H^+^]^−^
45	GT1(d18:1/14:0)	1034.9885	[M-2H^+^]^2−^
46	GT1(d18:1/18:0)	1063.0255	[M-2H^+^]^2−^
47	GT1(d18:1/20:3)	1074.0160	[M-2H^+^]^2−^
	GT1(d18:1/18:0)	1074.0160	[M-3H^+^+Na^+^]^2−^
48	GT1(d18:1/20:0)	1077.0427	[M-2H^+^]^2−^
49	GT1(d18:1/22:3)	1088.0338	[M-2H^+^]^2−^
	GT1(d18:1/20:0)	1088.0338	[M-3H^+^+Na^+^]^2−^
50	GT1(d18:1/22:0)	1091.0479	[M-2H^+^]^2−^
51	GT1(d18:1/24:1)	1115.0584	[M-3H^+^+Na^+^]^2−^
52	GM3(d18:0/16:0)	1151.7036	[M-H^+^]^−^
53	(CH_3_COO^−^)GalNAc-GT1(d18:1/16:2)	1179.0457	[M^−^-H^+^]^2−^
54	GA1(d18:1/18:0)	1235.7589	[M-H^+^ -H_2_O]^−^
55	GM3(d18:1/27:0)	1305.8889	[M-H^+^]^−^
56	GM3(d18:1/27:0)	1327.8499	[M-2H^+^+Na^+^]^−^
57	GD3(d18:1/16:0)	1442.8043	[M-H^+^]^−^
58	GM1(d18:1/14:0)	1488.8092	[M-H^+^]^−^
59	GM1(d18:1/22:0)	1600.9337	[M-H^+^]^−^
60	GD2(d18:1/17:0)	1659.8918	[M-H^+^]^−^
61	GalNAc-GM1(d18:1/8:1)	1745.9223	[M-H^+^]^−^
62	GD1(d18:1/16:0)	1807.9201	[M-H^+^]^−^

d-dihydroxylated sphingoid base; t-trihydroxylated sphingoid base.

**Table 4:** GGs and asialo-GG species from brain metastasis of LADC detected by (-) chip-nanoESI QTOF MS analysis of the native GG extract (adapted with permission from [160]).

*m/z* (Monoisotopic) Experimental	Molecular Ion	Proposed Structure
874.91	[M-H]^−^	LacCer(d18:1/17:0)
932.99	[M-H]^−^	LacCer(d18:0/21:0)
947.19	[M-H]^−^	LacCer(d18:0/22:0)
949.24	[M+2Na-3H]^−^	LacCer(d18:0/19:0)
963.90	[M-H]^−^	GM4(d18:0/14:0)
981.94	[M+Na-2H]^−^	GM4(d18:1/14:1)
983.87	[M+Na-2H]^−^	GM4(d18:1/14:0) or GM4(d18:0/14:1)
1122.23	[M-H]^−^	GA2(d18:0/20:0)
1138.15	[M-H]^−^	Fuc-GM4(d18:0/16:0) GA2(t18:0/20:0)
1150.17	[M-H]^−^	GA2(d18:1/21:0) GM3(d18:1/16:1)
1168.01	[M-H]^−^	GM3(t18:0/16:0)
1178.14	[M-H]^−^	GM3(d18:1/18:1)
1180.10	[M-H]^−^	GM3(d18:1/18:0)
1182.21	[M-H]^−^	GM3(d18:0/18:0)
1184.08	[M-H]^−^	*O*-Ac-GA1(d18:1/10:0)
1194.15	[M-H]^−^	GM3(d18:1/19:0) or GM3(d18:0/19:1)
1206.33	[M-H]^−^	GM3(d18:1/20:1) GA2(d18:0/26:0)
1222.19	[M-H]^−^	*O*-Ac-GM3(d18:1/18:0) GM3(d18:0/21:1) or GM3(d18:1/21:0)
1234.22	[M-H]^−^	GM3(d18:1/22:1) *O*-Ac- GM3(d18:1/19:1)
1248.18	[M-H]^−^	*O*-Ac-GM3(d18:1/20:1) GM3(d18:1/23:1)
1249.02	[M-H]^−^	GM3(d18:1/23:0)
1260.33	[M-H]^−^	GM3(d18:1/24:2)
1262.35	[M-H]^−^	GM3(d18:1/24:1)
1264.19	[M-H]^−^	GM3(d18:1/24:0)
1277.01	[M-H]^−^ [M-H]^−^ [M-H]^−^(-H_2_O)	*O*-Ac-GM3(d18:1/22:0) GM3(d20:1/23:1) GM3(d18:0/26:0)
1278.21	[M-H]^−^	GM3(d20:1/23:0) *O*-Ac-GM3(d18:1/22:0) or *O*-Ac-GM3(d18:0/22:1)
1289. 04	[M-H]^−^ [M-H]^−^	GM3(d18:1/26:2) or GM3(d18:2/26:1) GM3(d20:1/24:2)
1292.23	[M-H]^−^	GM3(d18:1/26:0) or GM3(d18:0/26:1)
1296.24	[M-H]^−^	Fuc-GM3(d18:1/16:1) *O*-Ac-GA1(d18:1/18:0) GA1(d18:0/21:0) or GA1(d18:0/21:0)
1405.21	[M+Na-2H]^−^	GM2(d18:1/18:0)
1420.80	[M-H]^−^	*O*-Ac-GM2(d18:2/18:2)
1435.21	[M+Na-2H]^−^	GD3(d18:1/14:1) or GD3(d18:0/14:2) or GD3(d18:2/14:0)
1441.19	[M-H]^−^	GD3(d18:1/16:1) or GD3(d18:0/16:2) or GD3(d18:2/16:0)
1471.28	[M-H]^−^	GD3(d18:1/18:0)
1493.23	[M+Na-2H]^−^	GD3(d18:1/18:0)
1515.29	[M+2Na-3H]^−^ [M-H]^−^	GD3(d18:1/18:0) or GD3(d18:0/18:1) GM1(d18:1/16:1) or GM1(d18:0/16:2) or GM1(d18:2/16:0) *O*-Ac-GD3(d18:0/18:0)
1516.01	[M+Na-2H]^−^ [M-H]^−^	GD3(d18:1/20:2) or GD3(d18:0/20:3) or GD3(d18:2/20:1) GM1(d18:2/16:0) or GM1(d18:1/16:1) GM1(d18:0/16:2)
1528. 16	[M-H]^−^	GD3(d18:0/22:0)
1542. 19	[M-H]^−^	GM1(d18:1/18:2) or GM1(d18:2/18:1) or GM1(d18:0/18:3)
1570.29	[M+2Na-3H]^−^ [M-H]^−^ [M+Na-2H]^−^	GD3(d18:1/22:0) or GD3(d18:0/22:1) GM1(d18:1/20:1) or GM1(d18:0/20:2) or GM1(d18:2/20:0) GD3(d18:0/24:2) or GD3(d18:1/24:1) or GD3(d18:2/24:0)
1598.09	[M-H]^−^	GM1(d18:0/22:2) or GM1(d18:1/22:1) or GM1(d18:2/22:0)
1612.17	[M+2Na-3H]^−^	GM1(d18:1/20:2)
1625.40	[M+2Na-3H]^−^ [M-H]^−^	GD3(d18:1/26:1) or GD3(d18:0/26:2) or GD3(d18:2/26:0) GM1(d18:1/24:2)
1627.41	[M+2Na-3H]^−^ [M-H]^−^	GD3(d18:0/26:1) or GD3(d18:1/26:0) GM1(d18:0/24:2) or GM1(d18:1/24:1) or GM1(d18:2/24:0)
1629.42	[M-H]^−^ [M-H]^−^	GM1(d18:0/24:1) or GM1(d18:1/24:0) di-*O*-Ac-GM1(d18:1/18:0)
1660.18	[M+3Na-4H]^−^	GM1(d18:1/22:3) or GM1(d18:0/22:4) or GM1(d18:2/22:2)
1675.23	[M+Na-2H]^−^(-H_2_O)	GD2 (d18:1/18:2)
1749.39	[M+Na-2H]^−^	GD2 (d18:1/22:1)
1767.28	[M-H]^−^(-H_2_O)	GT3 (d18:1/20:1)
1785.37	[M-H]^−^	*O*-Ac-GD2(d18:1/23:0) or *O*-Ac-GD2(d18:0/23:1)
1833.28	[M-H]^−^ [M-H]^−^	GT3(d18:0/23:0) *O*-Ac-GT3 (d18:0/20:0)
1861.24	[M-H]^−^ [M-H] (-H_2_O)	*O*-Ac-GT3-lactone(d18:0/22:0) *O*-Ac-GT3(d18:0/22:0)
1879.39	[M+Na-2H]^−^ [M-H]^−^(-H_2_O) [M-H]^−^	*O*-Ac-GT3 (d18:2/22:1) Fuc-GT3(d18:0/17:0) GT2(d18:1/12:1) or GT2(d18:2/12:0)
1909.03	[M-H]^−^	GD1 (d18:1/22:0)
1959.84	[M-H]^−^(-2H_2_O) [M-H]^−^	GT2(d18:0/20:0) GT2(d18:1/18:3) or GT2(d18:2/18:2)
1989.78	[M+Na-2H]^−^ [M-H]^−^	GT2(d18:0/18:0) GT2(d18:0/20:3) or GT2(d18:1/20:2) or GT2(d18:2/20:1)
1990.83	[M-H]^−^	GT2(d18:1/20:1) or GT2(d18:0/20:2) or GT2(d18:2/20:0)
2005.63	[M-H]^−^	Fuc-GD1(d18:1/20:2) *O*-Ac-GT2(d18:1/18:1)
2048.80	[M-H]^−^ [M-H]^−^(-H_2_O)	di-*O*-Ac-GT2(d18:0/18:0) GT1(d18:2/14:2) or GT1(d18:3/14:1)

**Table 5:** GG and asialo-GG species from normal brain detected by (-) chip-nanoESI QTOF MS analysis of the native GG extract (adapted with permission from [160]).

*m/z* (Monoisotopic) Experimental	Molecular Ion	Proposed Structure
708.38	[M-3H]^3−^	GT1(d18:1/18:0)
714.40	[M-3H]^3−^ [M+Na-4H]^3−^	GT1(t18:0/18:0) GT1(d18:1/18:2)
717.54	[M-3H]^3−^	GT1(d18:1/20:0) or GT1(d18:0/20:1)
735.12	[M-2H]^2−^	GD3(d18:1/18:0) or GD3(d18:0/18:1)
756.30	[M-2H]^2−^	*O*-Ac-GD3(d18:1/18:0)
771.93	[M-2H]^2−^	GM1(d18:0/18:1) or GM1(d18:1/18:0)
822.06	[M+Na-4H]^3−^	GQ1(d18:1/20:0) or GQ1(d18:0/20:1)
835.69	[M-2H] ^2−^	GD2(d18:1/18:1) or GD2(d18:0/18:2) or GD2(d18:2/18:0)
844.69	[M-2H]^2−^	*O*-Ac-GD2(d18:0/16:0)
850.22	[M-2H]^2−^	GD2 (d18:1/20:0)
863.21	[M-2H]^2−^ [M-2H]^2−^ [M+Na-3H]^2−^	Fuc-GM1(d18:1/22:2) or Fuc-GM1(d18:0/22:3) or Fuc-GM1(d18:2/22:1) GD2(d18:0/22:3) or GD2(d18:1/22:2) or GD2(d18:2/22:1) GD2(d18:0/20:0)
877.88	[M-2H]^2–^	GD2(d18:1/24:1)
885.78	[M-2H]^2−^	*O*-Ac-GD2(d18:1/22:0) or *O*-Ac-GD2(d18:0/22:1)
890.76	[M+Na-3H]^2−^	GT3(d18:1/18:1)
905.11	[M+Na-3H]^2−^	GT3(d18:1/20:0)
917.44	[M-2H]^2−^	GD1(d18:1/18:0) or GD1(d18:0/18:1)
924.76	[M-2H]^2–^ [M+2Na-4H]^2−^	GD1(d18:1/19:0) or *O*-Ac-GT3(t18:1/20:0) or *O*-Ac-GT3(d18:0/20:1)
931.45	[M-2H]^2−^	GD1(d18:1/20:0) or GD1(d18:0/20:1)
940.46	[M-2H]^2–^ [M+2Na-4H]^2−^	GD1(t18:0/20:0) GD1 (d18:1/18:0) or GD1(d18:0/18:1)
945.50	[M-2H]^2−^	GD1(d18:1/22:0)
952.50	[M-2H]^2−^	*O*-Ac-GD1 (d18:1/20:0) or *O*-Ac-GD1 (d18:0/20:1)
968.34	[M-3H]^3−^	GH2(d18:1/24:0) or GH2(d18:0/24:1)
991.27	[M+Na-3H]^2−^	GT2(d18:1/18:2) or GT2(d18:0/18:3) or GT2(d18:2/18:3)
1005.28	[M+2Na-4H]^2−^	GT2 (d18:0/18:0)
1019.36	[M-2H]^2–^	GalNAc-GD1(d18:0/18:0)
1024.68	[M-2H]^2–^ [M+2Na-4H]^2−^	di-*O*-Ac-GT2 (d18:1/18:0) *O*-Ac-GT2 (d18:1/18:1)
1033.94	[M+2Na-3H]^−^	GM4 (d18:1/16:0) or GM4 (d18:0/16:1)
1042.51	[M-2H]^2^	GT1 (t18:1/14:1) or GalNAc-GD1(t18:0/20:0)
1046.46	[M+Na-3H]^2−^	GT1(d18:1/14:0) or GT1(d18:0/14:1)
1049.51	[M-2H]^2−^	GT1(d18:1/16:0) or GT1(d18:0/16:1)
1059.28	[M+Na-3H]^2−^	GT1(d18:1/16:1)
1063. 35	[M-2H]^2−^	GT1(d18:1/18:0) or GT1(d18:0/18:1)
1074.05	[M+Na-3H]^2−^	GT1(d18:1/18:0)
1077.37	[M-2H]^2−^	GT1(d18:1/20:0)
1096.81	M-2H]^2−^ [M+Na-3H]^2−^	*O*-Ac-GT1(d18:1/20:1) or *O*-Ac-GT1(d18:0/20:2) or *O*-Ac-GT1(d18:2/20:0) GT1(t18:0/20:0)
1110.36	[M-2H]^2−^	*O*-Ac-GT1(d18:1/22:2)
1118.56	[M-H]^−^	GM4(t18:1/24:0)
1180.09	[M-H]^−^	GM3(d18:1/18:0) or GM3(d18:0/18:1)
1228.61	[M-H]^−^ [M+Na-2H]^−^	GA1(d18:0/16:0) GM3(d18:1/20:1) or GM3(d18:0/20:2)
1232.12	[M-H]^−^ [M+Na-2H]^−^	GM3(d18:1/22:2) or GM3(d18:0/22:3) or GM3(d18:2/22:1) GM3(d18:0/20:0)
1240.86	[M+Na-3H]^2−^	*O*-Ac-GQ1(d18:1/18:0)
1252.19	[M-H]^−^	*O*-Ac-GM3(d18:0/20:0) GM3 (d18:0/23:0)
1264.12	[M-H]^−^	di-*O*-Ac-GM3(d18:1/18:0)
1284.36	[M+Na-2H]^−^	GM3(d18:1/24:1)
1382.87	[M-H]^−^	GM2(d18:1/18:0) or GM2(d18:0/18:1)
1410.19	[M-H]^−^	GM2(d18:1/20:0) or GM2(d18:0/20:1)
1467.86	[M-H]^−^ [M+Na-2H]^−^	GD3(d18:1/18:2) GD3(d18:0/16:0)
1492.89	[M+Na-2H]^−^	GD3(d18:1/18:0)
1509.71	[M-H]^−^	*O*-Ac-GD3(d18:1/18:1) Fuc-GM2-lactone (d18:1/18:1)
1513.29	[M-H]^−^	*O*-Ac-GD3(d18:1/18:0)
1517.30	[M-H]^−^ [M+Na-2H]^−^ [M+Na-2H]^−^	GM1(d18:1/16:0) GD3(d18:2/20:2) Fuc-GM2(d18:2/16:2)
1537.26	[M+Na-2H]^−^	GM1(d18:1/16:1)
1541.21	[M+2Na-3H]^−^	GD3(d18:1/20:0)
1544.92	[M-H]^−^	GM1(d18:1/18:0) or GM1(d18:0/18:1)
1561.36	[M-H]^−^	*O*-Ac-GM1(d18:0/16:0)
1566.68	[M+Na-2H]^−^	GM1(d18:1/18:0) or GM1(d18:0/18:1)
1572.92	[M-H]^−^	GM1(d18:1/20:0) or GM1(d18:0/20:1)
1589.28	[M-H]^−^	Fuc-GD3(d18:1/16:0)
1594.16	[M+Na-2H]^−^	GM1(d18:1/20:0)
1600.23	[M-H]^−^	GM1(d18:0/22:0)
1629.32	[M-H]^−^	di-*O*-Ac-GM1(d18:1/18:0)
1648.32	[M-H]^−^	GD2(d18:0/16:0)
1656.88	[M-H]^−^	GD2-lactone (d18:1/18:0)
1663.16	[M+Na-2H]^−^	GD2(d18:2/16:2)
1674.33	[M-H]^−^	GD2(d18:1/18:0) or GD2(d18:0/18:1)
1690.95	[M-H]^−^	Fuc-GM1(d18:1/18:0)
1701.42	[M-H]^−^	GD2(d18:1/20:0) or GD2(d18:0/20:1)
1709.36	[M+Na-2H]^−^	*O*-Ac-GD2 (d18:1/16:1)
1716.91	[M-H]^−^	Fuc-GM1 (d18:1/20:1)
1718.48	[M-H]^−^	Fuc-GM1 (d18:1/20:0)
1729.62	[M-H]^−^	GD2(d18:1/22:0) or GD2(d18:0/22:1)
1741.27	[M+Na-2H]^−^	Fuc-GM1 (d18:0/20:0)
1746.38	[M+2Na-3H]^−^	GD2(d18:0/20:0)
1775.57	[M+Na-2H]^−^	GD2(d18:2/24:3)
1787.76	[M+2Na-3H]^−^	Fuc-GM1(d18:1/22:1)
1796.50	[M+Na-2H]^−^	Fuc-GM1(d18:1/24:0)
1803.64	[M-H]^−^	*O*-Ac-GT3(d18:1/18:1)
1835.91	[M-H]^−^	GD1(d18:1/18:0) or GD1(d18:0/18:1)
1857.59	[M+Na-2H]^−^	GD1(d18:0/18:0)
1863.61	[M-H]^−^	GD1(d18:1/20:0)
1872.68	[M+Na-2H]^−^	GD1(d18:1/19:0)
1879.91	[M+2Na-3H]^−^	GD1(d18:1/18:0)
1886.98	[M-H]^−^	Fuc-GT3-lactone(d18:1/18:2) Fuc-GT3-lactone(d18:0/18:3) Fuc-GT3-lactone(d18:2/18:1)
1896.06	[M+2Na-3H]^−^	GD1(d18:0/19:0)
1901.71	[M+2Na-3H]^−^	*O*-Ac-GT3(d18:1/22:1)
1909.43	[M-H]^−^	GT2(d18:0/14:0) or GT2(d18:0/14:1)
1915.78	[M-H]^−^	GD1(d18:1/24:2)
1925.77	[M+Na-2H]^−^	GD1(d18:1/23:1)
1937.80	[M+Na-2H]^−^	GD1(d18:1/24:2) or GD1(d18:0/24:3) or GD1(d18:2/24:1)
1964.84	[M-H]^−^	GT2(d18:0/18:0)
1982.55	[M-H]^−^	Fuc-GD1(d18:1/18:0)
2004.61	[M-H]^−^	Fuc-GD1(d18:1/20:2)
2010.78	[M+Na-2H]^−^	GT2(d18:1/20:2) or GT2(d18:0/20:3) or GT2(d18:2/20:1)
2032.63	[M-H]^−^ [M+2Na-3H]^−^	*O*-Ac-GT2(d18:1/20:1) or *O*-Ac-GT2(d18:0/20:2) or *O*-Ac-GT2(d18:2/20:0) GT2(d18:1/20:2) or GT2(d18:0/20:3) or GT2(d18:2/20:1)
2049.75	[M-H]^−^	di-*O*-Ac-GT2 (d18:1/18:0)
2059.78	[M+Na-2H]^−^	Fuc-GD1(d18:1/22:0)
2076.85	[M-H]^−^	GQ3(d18:1/20:2) or GQ3(d18:0/20:3) or GQ3(d18:2/20:1)
2105.78	[M-H]^−^(-H_2_O)	GT1(d18:1/18:2)
2165.56	[M-H]^−^ [M+Na-2H]^−^	*O*-Ac-GT1(d18:1/18:2) or *O*-Ac-GT1(d18:0/18:3) or *O*-Ac-GT1(d18:2/18:1) GT1(t18:1/18:0) *O*-Ac-GT1(d18:1/16:0)
2172.04	[M+2Na-3H]^−^	O-Ac-GT1(t18:1/14:1)
2187.62	[M+Na-2H]^−^(-H_2_O) [M+Na-2H]^−^	GT1(d18:1/22:0) GT1(d18:1/21:1)
2198.08	[M+2Na-3H]^−^	GT1(d18:1/20:0)
2215.78	[M+Na-2H]^−^	*O*-Ac-GT1(d18:1/20:2)
